# Photochemical H_2_ activation by an Zn–Fe heterometallic: a mechanistic investigation†

**DOI:** 10.1039/d3sc05966a

**Published:** 2023-12-14

**Authors:** Marina Perez-Jimenez, Mark R. Crimmin

**Affiliations:** a Department of Chemistry, Molecular Sciences Research Hub, Imperial College London 82 Wood Lane, White City London W12 0Z UK m.crimmin@imperial.ac.uk

## Abstract

Addition of H_2_ to a Zn–Fe complex was observed to occur under photochemical conditions (390 or 428 nm LED) and leads to the formation of a heterometallic dihydride complex. The reaction does not occur under thermal conditions and DFT calculations suggest this is an endergonic, light driven process. Through a combined experimental and computational approach, the plausible mechanisms for H_2_ activation were investigated. Inhibition experiments, double-label cross-over experiments, radical trapping experiments, EPR spectroscopy and DFT calculations were used to gain insight into this system. The combined data are consistent with two plausible mechanisms, the first involving ligand dissociation followed by oxidative addition of H_2_ at the Fe centre, the second involving homolytic fragmentation of the Zn–Fe heterometallic and formation of radical intermediates.

## Abstract 2

Addition of H_2_ to a Zn–Fe complex was observed to occur under photochemical conditions (390 or 428 nm LED) and leads to the formation of a heterometallic dihydride complex.

## Graphical abstract

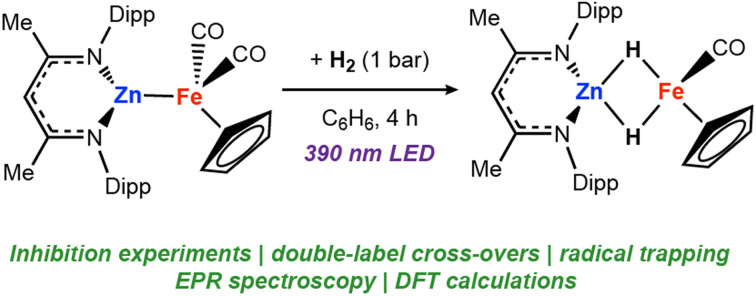

## Introduction

In the past few years there has been growing interest in using heterometallic complexes for H_2_ activation. Transition metal complexes supported by Lewis-acidic B,^1–3^ Al,^4^ Zn,^5,6^ Mg,^7,8^ and Sn^9,10^ ligands have all been documented to react with H_2_ under thermal conditions. Despite the rapid growth of this area, examples of photolytic H_2_ splitting using these types of heterometallic complexes are rare. To the best of our knowledge, photolytic reactivity is limited to two examples involving the addition of H_2_ to Ru–Zn and Ru–Al heterometallic complexes.^11,12^

These findings contrast the rich chemistry of single-site transition metal complexes, where examples of photochemical H_2_ activation are more common. Under photolytic conditions, 18-electron transition metal carbonyl complexes are prone to ligand dissociation to generate coordinatively unsaturated intermediates that can react with H_2_. For example, irradiation of mixtures [M(CO)_6_] and H_2_ at low temperature leads to the formation of the corresponding dihydrogen complexes [M(CO)_5_(η^2^-H_2_)] (M = Cr, W).^13^ In contrast, photochemical reaction of [Ru(CO)_3_(PPh_3_)_2_] with H_2_ leads to oxidative addition to form [Ru(CO)_2_(PPh_3_)_2_(H)_2_].^14^

Bimetallic transition metal carbonyl complexes such as [Ru(η^5^-C_5_H_5_)(CO)_2_]_2_ also react with H_2_ under photochemical conditions.^15^ In these cases, photolysis can promote either dissociation of CO or homolysis of the metal–metal bond forming 17-electron species.^16–18^ As a result, a diverse set of mechanistic pathways involving both neutral and radical intermediates are possible for H_2_ splitting.^19,20^

In this paper, we report the reaction of a heterometallic Zn–Fe carbonyl complex with H_2_ under photochemical conditions; this is an uphill, light-driven reaction (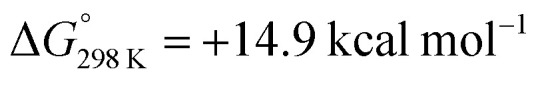; Δ*H*° = +17.5 kcal mol^−1^). Using a combined experimental (inhibition, cross-over, radical trapping, EPR spectroscopy) and computational (DFT) approach, we present a detailed investigation into the plausible pathways of H_2_ activation at the heterometallic complex. We conclude that the most likely pathways involve (i) a closed-shell mechanism involving CO dissociation and subsequent oxidative addition at Fe, and (ii) an open-shell pathway involving homolysis of the Zn–Fe bond to form radical intermediates capable of reacting with H_2_. Our findings provide rare insight into a light-driven process that operates at a heterometallic complex,^21–24^ and add to the growing examples that invoke the formation of radical intermediates.

## Results and discussion

### Photochemical H_2_ activation

**Scheme 1:**
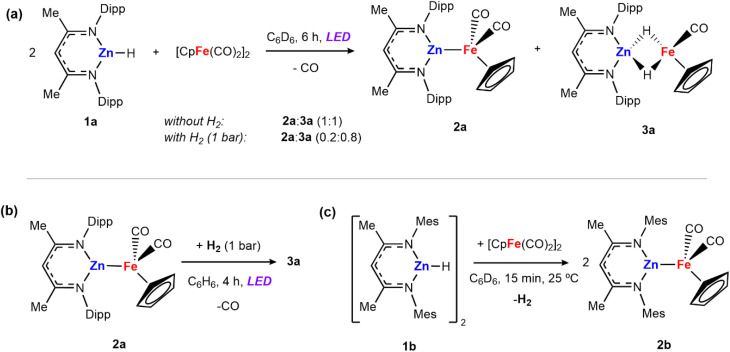
(a) Reactions of 1a with [Fe(η^5^-C_5_H_5_)(CO)_2_]_2_ to form a mixture of compounds 2a and 3a; (b) reaction of 2a with dihydrogen under photochemical conditions; (c) reaction of 1b with [Fe(η^5^-C_5_H_5_)(CO)_2_]_2_ to form 2b and release H_2_.

Irradiation of [Zn(H){CH(CMeNAr)_2_}] (1a, Ar = 2,6-^i^Pr_2_C_6_H_3_) and 0.5 equiv. of [Fe(η^5^-C_5_H_5_)(CO)_2_]_2_ for 6 hours with either a 390 or 428 nm LED lamp (40 W) leads to the formation of a mixture of heterometallic species [(η^5^-C_5_H_5_)(CO)Fe–Zn{CH(CMeNAr)_2_}] (2a) and [(η^5^-C_5_H_5_)(CO)Fe(H)_2_Zn{CH(CMeNAr)_2_}] (3a) in a 1 : 1 ratio (Scheme 1a). The mixture of 2a and 3a in benzene-*d*_6_ showed no changes over time at 25 °C, or upon heating to 100 °C. Both 2a and 3a are diamagnetic and can be assigned formal oxidation states of Fe(0) and Fe(ii) respectively. Complex 2a is a Zn–Fe adduct which has been previously reported by Mankad and co-workers,^25^ while complex 3a features two bridging hydride groups linking the Zn and Fe centres. Formation in a 1 : 1 ratio conserves the reaction stoichiometry. Generation of 3a could be considered to derive from: (i) 1a reacting with [Fe(η^5^-C_5_H_5_)(CO)_2_]_2_ to form 2a and [Fe(η^5^-C_5_H_5_)(H)(CO)_2_]^26–28^ as a transient intermediate which is then trapped by a second equiv. of 1a to form 3a or (ii) addition of *in situ* generated H_2_ across the Zn–Fe bond of 2a under photochemical conditions. To test this second potential mechanism involving photochemical H_2_ splitting, 1a and [Fe(η^5^-C_5_H_5_)(CO)_2_]_2_ were reacted with dihydrogen (1 bar) under LED light. A change in the ratio of the two products was observed reaching a 0.2 : 0.8 (2a : 3a) mixture in 6 hours (Scheme 1a).

Independently prepared samples of 2a also react with H_2_ under photochemical conditions (blue LED lamp, 40 W, 390 nm) to form 3a in 55% yield by ^1^H NMR spectroscopy after 4 h (Scheme 1b). No reaction occurred under thermal conditions (24 h at 80 °C). While this experiment cannot rule out the direct formation of 3a from 1a and [Fe(η^5^-C_5_H_5_)(CO)_2_]_2_ it suggests that 2a is capable of activating H_2_ under photolysis. The reaction was monitored by ^1^H NMR spectroscopy to quantify the formation of 3a, as well as minor products 1a and [Fe(η^5^-C_5_H_5_)(CO)_2_]_2_ which are formed alongside the product. When employing either 390 or 428 nm LED Kessil lamps, longer exposures times led to decreases in the yield of 3a.

UV-vis spectra were recorded for 2a and the dihydride product 3a. Both species present similar absorption bands with a maximum absorption peak around 350 nm. The light absorption of both starting material and product at the same wavelength might explain the challenge of achieving higher yields and the instability of 3a during photolysis. From all the conditions tested, the best results obtained were: 1 bar H_2_, benzene solvent, 4 hours irradiation using a 390 LED lamp (3a, 45% isolated yield or 55% NMR yield). Experiments using *D*_2_ showed 92% deuterium incorporation in 3a, demonstrating that the hydride groups come from dihydrogen, rather than from other possible sources *i.e.* reaction with the solvent.

The propensity for H_2_ activation in this system proved sensitive to the precise sterics of the ligand environment on zinc and reaction of [Zn(H){CH(CMeNMes)}] (1b, Mes = 2,4,6-Me_3_C_6_H_2_), bearing smaller aryl flanking groups, with [Fe(η^5^-C_5_H_5_)(CO)_2_]_2_ led to the immediate formation of ([(η^5^-C_5_H_5_)(CO)Fe–Zn{CH(CMeNMes)_2_}] (2b) with elimination of dihydrogen at 25 °C (Scheme 1c). A control reaction was conducted in which 2b was exposed to H_2_ in the presence of light. However, no hydride-containing products were detected at short reaction times (1 bar H_2_, 1–6 hours) or long exposure times (24 hours) and under high H_2_ pressures (4 bar).

### Characterisation and bonding analysis

**Fig. 1:**
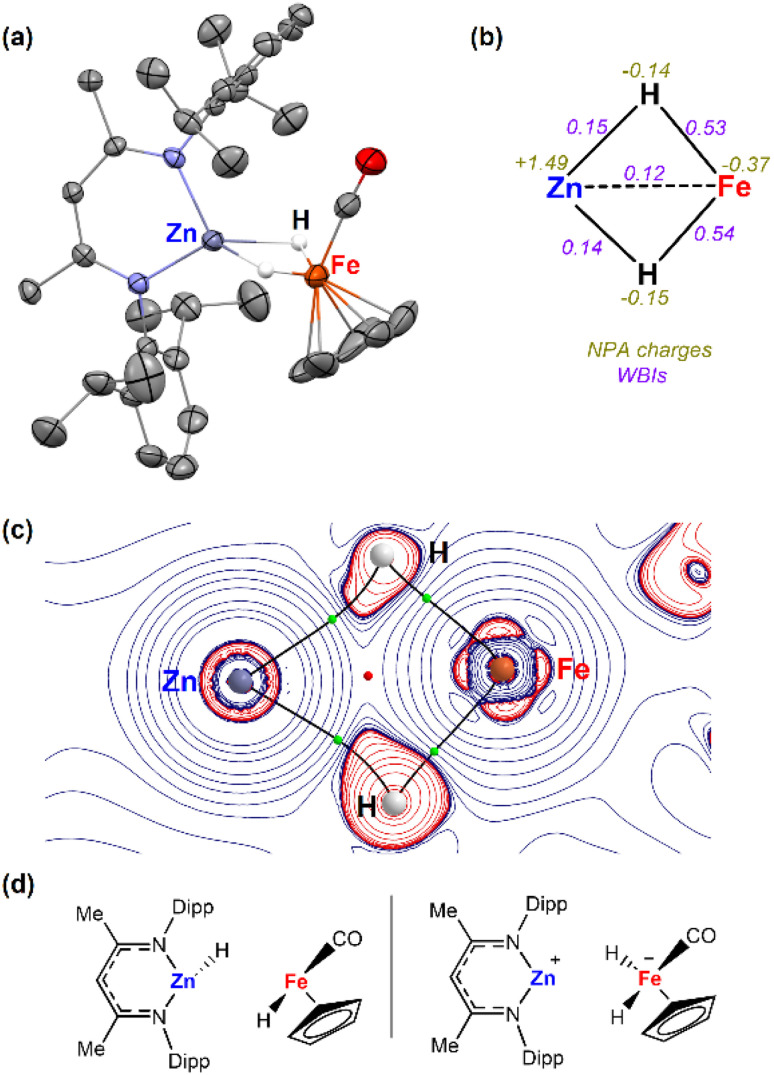
(a) Single-crystal X-ray structures of complex 3a (50% probability ellipsoids) hydrogen atoms were removed from the ORTEP structure for clarity except the bridging hydrides; (b) NBO, (c) QTAIM molecular graphs of 3a. Showing key bond critical points (BCPs, green spheres) and ring critical points (RCPs, red spheres), and (d) fragmentations analysed by ETS-NOCV.

Complex 3a was characterised by X-ray crystallography, along with multinuclear NMR and IR spectroscopy. The ^1^H NMR spectrum shows characteristic signals in the hydride region at *δ* = −15.98 ppm (2*H*). The IR also exhibited bands at 1780 and 1810 cm^−1^ for the bridging hydrides and at 1948 cm^−1^ for the CO group. The structure was confirmed after obtaining crystals suitable for X-ray diffraction studies from the reaction mixture of 1a and [Fe(η^5^-C_5_H_5_)(CO)_2_]_2_ (Fig. 1a). Two molecules co-crystallized in the same unit cell, one corresponding to the Zn–Fe adduct (2a) and the other to the dihydride complex (3a). The Zn–Fe distance of 2.384(1) Å in complex 2a is elongated to 2.404(1) Å in complex 3a due to the presence of two bridging hydrides (Fig. 1a). The hydrogen atoms were located in the Fourier difference map during X-ray experiments and their positions confirmed by DFT calculations (Table S3†).

**Fig. 2:**
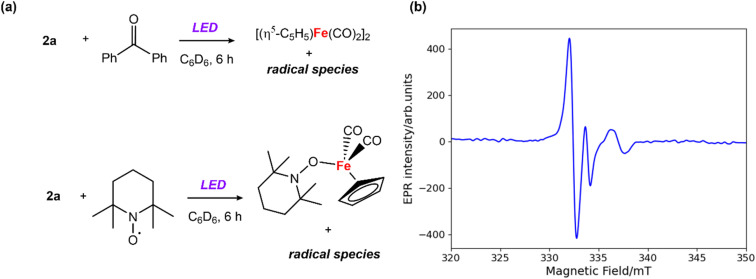
(a) Reactions between 2a and benzophenone/TEMPO; (b) CW X-band EPR spectrum recorded in benzene solution at ambient temperature; microwave frequency, 9.44667 GHz; field modulation amplitude, 0.25 mT.

Natural Bonding Orbital (NBO) calculations of complex 3a provided information on the bonding of the heterometallic motif (Fig. 1b). Based on the analysis the hydrides are best considered as bridging ligands which participate in 3-centre 2-electron bonding with Zn and Fe. There is limited support for a significant Zn⋯Fe interaction. Wiberg Bond Indices (WBI) for the Fe–H bonds are higher than those of the corresponding Zn–H bonds. In comparison the Zn⋯Fe WBI (0.12) of 3a is lower than those between the metals and hydrides and lower compared to the Zn⋯Fe WBI in 2a (0.31). NPA charges are negative on the Fe and H atoms but electropositive on Zn (Fig. 2b). The calculations suggest that the hydrides form the most significant covalent interaction with Fe rather than Zn. QTAIM analysis were also undertaken on 3a showing four bond critical points (bcps) between each of the H atoms and Fe or Zn atoms but not between the metals themselves (Fig. 1c). A ring critical point (rcp) was found in the middle of the four membered ring. ETS-NOCV calculations were also performed on 3a. The molecule was separated into two neutral metal hydride fragments, [Fe]–H and [Zn]–H. A total interaction energy Δ*E*_ORB_ = −80.4 kcal mol^−1^ was obtained, with the two principal contributions corresponding to donation from the [Zn]–H bond towards the [Fe] centre (Δ*ρ*_1_ = −41.1 kcal mol^−1^) and donation from the [Fe]–H bond to the [Zn] centre (Δ*ρ*_2_ = −25.8 kcal mol^−1^). A second option was also considered (Fig. 1d), dividing the molecule into [Zn]^+^ and [Fe]H_2_^−^ fragments. In this case, a similar total interaction energy of Δ*E*_ORB_ = −93.5 kcal mol^−1^ was obtained, with the main contribution (Δ*ρ*_1_ = −43.6 kcal mol^−1^) corresponding to the donation from the two [Fe]–H bonds to the Zn atom.

### Plausible mechanism(s) of H_2_ activation

**Scheme 2:**
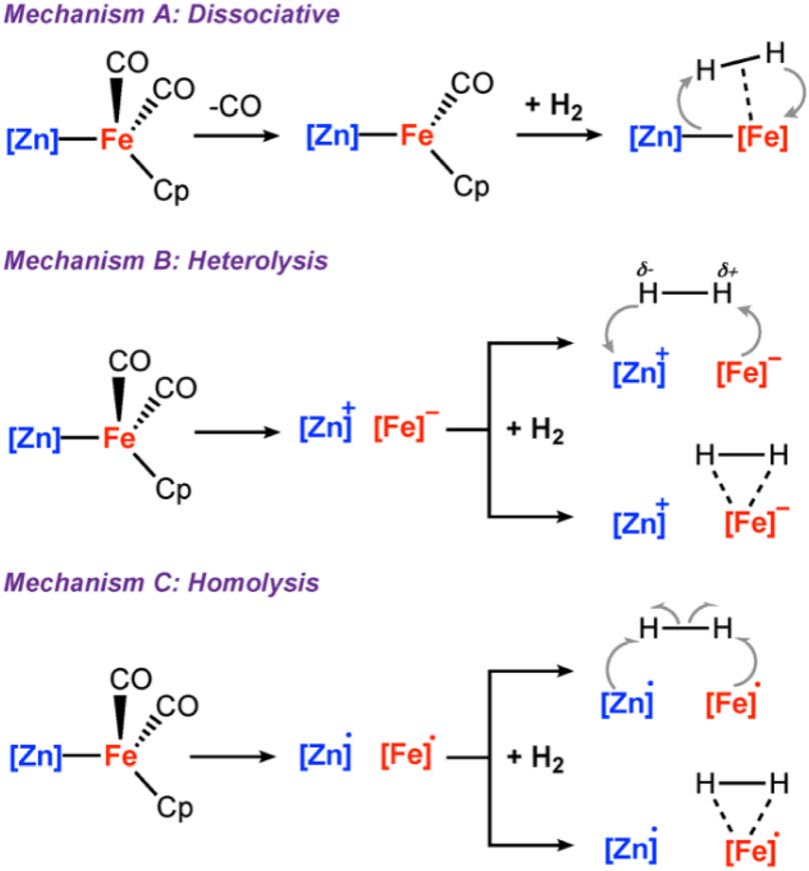
(A) ligand dissociation followed by H_2_ addition; (B) metal–metal heterolysis; (C) metal–metal homolysis.

Although H_2_ activation with heterometallic complexes consisting of both transition and main group metals is gaining increasing attention, to date most examples involve thermal conditions. The discovery of a new photolytic reaction in this area, raises questions as to how the mechanism proceeds under photochemical conditions. The overall reaction of 2a + H_2_ to form 3a is calculated to be endergonic with 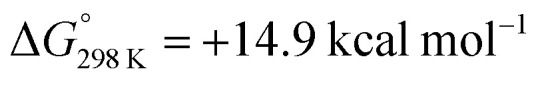 and Δ*H*° = +17.5 kcal mol^−1^, consistent with a light-driven process. 2a contains an Fe site with a formal 18-electron count that does not to react in the absence of light. Irradiation is expected to result in this complex accessing an excited state through changes in geometry, ligand dissociation, or fragmentation of the metal–metal bond. Several plausible pathways for photochemical H_2_ activation were considered (Scheme 2). These included: (*Mechanism A*) ligand dissociation from 2a to generate a 16-electron coordinatively unsaturated intermediate that reacts with H_2_ by either direct addition to Fe or across the Fe–Zn bond; (*Mechanism B*) heterolysis of the metal–metal bond to generate zwitterionic fragments that react with H_2_ by a closed-shell pathway; (*Mechanism C*) homolysis of the metal–metal bond to form a radical pair that reacts with H_2_ by an open-shell pathway. Experiments and calculations were undertaken to try and draw conclusions on the most likely possibilities.

*Mechanism A*: during the reaction with H_2_, CO must dissociate from 2a to ultimately form the product 3a. Dissociation could potentially occur prior to, or after, the H_2_ splitting step. The order of these events was probed through inhibition and isotope labelling experiments. The addition of 1 : 1 mixture of CO : H_2_ to a solution of 2a resulted in a complete inhibition of H_2_ activation, even under the exposure of LED light over 24 hours. Reaction of 2a with ^13^CO demonstrated that ligand exchange was possible at 25 °C after 20 h in the absence of light. This reaction was quicker under photochemical conditions and complete in 2 h on irradiation with 390 nm LEDs. These experiments support the facile and reversible dissociation of CO from 2a under photochemical conditions and based on these findings it is likely this occurs before H_2_ activation step. Dissociation of CO from 2a would generate a coordinatively unsaturated 16-electron intermediate that has the potential to directly react with H_2_ by an oxidative addition step.^29^

**Scheme 3:**
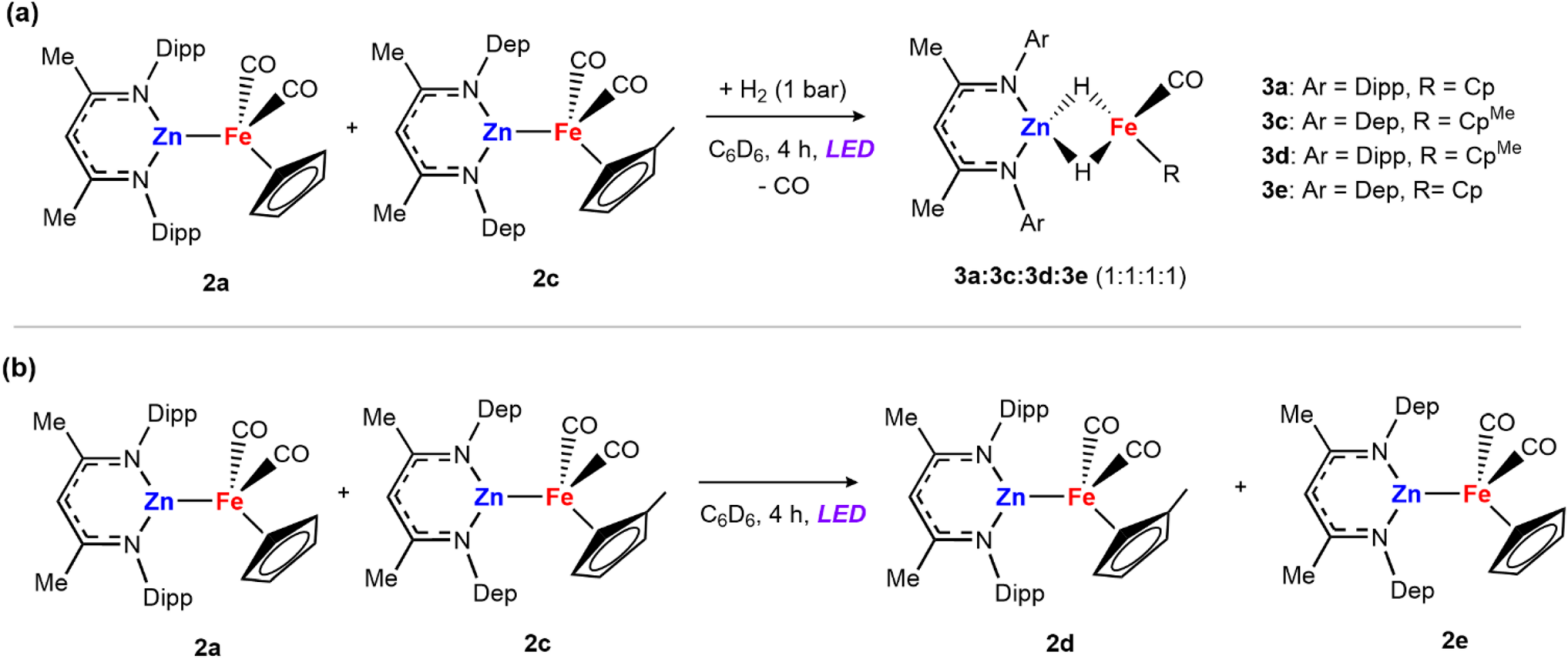
Cross-over experiments: (a) mixture of 2a and 2c in the presence of dihydrogen; (b) reaction between 2a and 2c in the absence of H_2_.

*Mechanism B and C*: the alternative mechanisms for H_2_ activation with 2a both involve fragmentation of the heterometallic into monomeric units. To probe this possibility, we conducted double-label cross-over experiments. Complex 2c was prepared by salt metathesis reaction of Na[Fe(η^5^-C_5_H_4_CH_3_)(CO)_2_]_2_ with [ZnCl{CH(CMeN-2,6-Et_2_C_6_H_3_)_2_}] and exposed to H_2_ to generate the corresponding dihydride species 3c. Then, a mixture of 2a and 2c were dissolved in benzene-*d*_6_ and the mixture was exposed to 1 bar of dihydrogen and the 390 nm LED lamp. After 4 hours, an equimolar ratio of the four hydride species 3a, 3c, 3d and 3e was detected by ^1^H NMR experiments (Scheme 3a).

The formation of cross-over products is consistent with fragmentation of the heterometallic under the reaction conditions. However, the independent mixture of 3a and 3c, also generated the mixed hydrides 3d and 3e, under the same photochemical conditions, meaning that the fragmentation of the dihydride could also account for the products observed. To further support that fragmentation comes from the initial adducts *via* metal–metal bond cleavage, a solution of 2a and 2c was treated under the same reaction conditions in the absence of the H_2_ atmosphere (Scheme 3b). After 4 hours under LED light the mixed iron product [Fe_2_(η^5^-C_5_H_4_CH_3_)(η^5^-C_5_H_5_)(CO)_4_] was detected, alongside 2d and 2e. Adduct 2e was prepared independently *via* the salt metathesis route and spectroscopic data match those of the cross-over experiment. These cross-over experiments suggest that breaking of the metal–metal bond can occur under photochemical conditions.

**Scheme 4:**
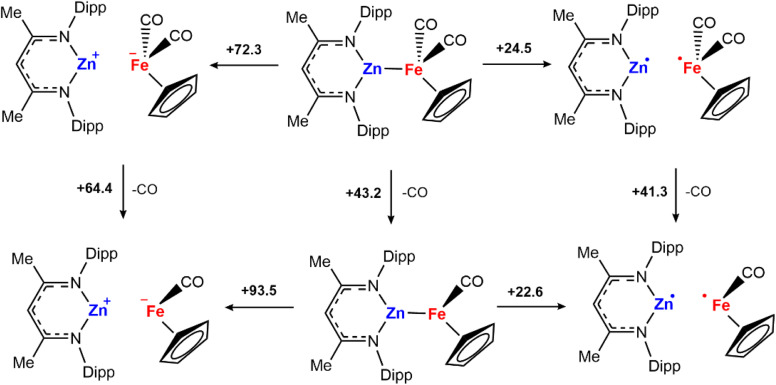
CO and metal–metal bond dissociation Gibbs free energies at 298 K in kcal mol^−1^. G09: BP86/def2TZVP (C, H, N, O)/SDDAll (Fe, Zn)//BP86/6-31g**(C, H)/6-311+g*(N, O)/SDDAll (Fe, Zn). Solvent corrections (benzene, epsilon = 2.2706) which were modelled using the polarized continuum model (PCM).

To further understand whether metal–metal bond cleavage could occur by heterolytic or homolytic pathways, a series of DFT calculations were undertaken. The energies for the homolytic and heterolytic cleavage of the metal–metal bond were calculated for 2a (Scheme 4). These steps were evaluated for structures with CO associated and CO dissociated. While barriers to cleavage are universally high, the energies for the homolytic dissociation were significantly lower (<25 kcal mol^−1^) compared to the heterolysis. The dissociation energies were calculated without including semiempirical dispersion corrections, as invoking these resulted in deviations from experimental results and over-estimated binding energies.^30,31^ Furthermore, similar barriers for homolysis of Al–Fe bonds have been calculated by Mankad and coworkers for a closely related system.^32^

The calculations suggest that the results of cross-over experiments are most likely due to homolysis rather than heterolysis of the metal–metal bond. To further probe this hypothesis, reactions with H_2_ were conducted with a series of different solvents. Heterolysis results in charge separation and as such, it might be expected that more polar solvents would facilitate this pathway increasing yields at shorter reaction times. The reaction of H_2_ with 2a in different solvents (benzene, *ε* = 2.3; THF, *ε* = 7.6; fluorobenzene, *ε* = 5.4) during the same reaction times (6 h) led to similar NMR yields for formation of 3a.^33^ Given these results, we consider that the homolytic metal–metal bond cleavage to form radical intermediates is more probable to occur compared to the heterolytic version.

EPR experiments were performed to probe the potential formation of radical intermediates. Addition of 1 equiv. of benzophenone to a solution of 2a in benzene and 6 hours exposure to LED light led to a distinctive signal in the X-band EPR spectrum (*g*_iso_ = 2.031) at 25 °C (Fig. 2b). A similar EPR signal was observed from the reaction the analogous Zn–Zn bonded complex [Zn_2_{CH(CMeN-2,4,6-Me_3_C_6_H_2_)_2_}_2_] with benzophenone under the same photochemical conditions (Fig. S17†). The short lifetime of the radical species, presumed to be a benzophenone radical anion coordinated to zinc, prevented its isolation and characterisation. While attempts to crystallise this species from the reaction mixture resulted in isolation of the starting material, a closely related magnesium analogue has been prepared by Jones and coworkers.^34^ EPR studies on the Mg-benzophenone radical reported by Jones^34^ (*g*_iso_ = 2.004) and the Al-benzophenone radical by Mankad^32^ (*g*_iso_ = 2.006) present similar *g* values to our measurements. During these experiments formation of [Fe(η^5^-C_5_H_5_)(CO)_2_]_2_ was observed by ^1^H NMR spectroscopy. In contrast, the addition of 1 equiv. of 2,2,6,6-tetramethylpyridine 1-oxyl (TEMPO) to 2a under the same conditions led to formation of [Fe(η^5^-C_5_H_5_)(CO)_2_(TEMPO)] (detected by ^1^H NMR experiments),^35^ and inhibited the formation of [Fe(η^5^-C_5_H_5_)(CO)_2_]_2_ (Fig. 2a).^36^ The EPR measurements and radical trapping experiments support homolysis of the Zn–Fe bond under photolytic conditions, with clear evidence for the formation and trapping of Fe-based radicals.

In combination, the cross-over experiments, calculations, solvent effects, and EPR data suggest that while heterolytic fragmentation of 2a is unlikely under the reaction conditions, homolytic cleavage leading to a radical pathway remains a distinct mechanistic possibility for H_2_ activation. Following homolysis of the metal–metal bond, the addition of H_2_ could potentially occur through either a single-site mechanism involving addition at the Fe fragment to generate a Fe-dihydride radical, which recombines with the zinc radical or a radical pair mechanism in which both metal radicals react with H_2_ in a concerted manner (Scheme 2C). The thermodynamics for these options were calculated by DFT, both are exergonic and feasible.

## Conclusions

In summary, we report a rare example of photochemical H_2_ activation with a Zn–Fe heterometallic complex. Through a combined experimental, spectroscopic, and computation approach we have interrogated plausible mechanisms for bond breaking of H_2_. We concluded that there is no unequivocal evidence that supports a single mechanism for the reaction. The combination of experimental and computational data suggests two mechanisms are possible and may operate simultaneously. One involves photochemical dissociation of CO from the Zn–Fe complex to form a coordinatively saturated intermediate that can react with H_2_*via* oxidative addition and the other involves homolytic cleavage of the Zn–Fe bond to form a pair of radicals that react directly with H_2_ through a radical pathway.

## Data availability

Data are available within the ESI.† Crystallographic data can be obtained *via*https://www.ccdc.cam.ac.uk/data_request/cif, or by emailing data_request@ccdc.cam.ac.uk.

## Author contributions

MPJ conducted all the experimental and computational work. All authors were involved in preparing the manuscript.

## Conflicts of interest

There are no conflicts to declare.

## Supplementary Material

SC-015-D3SC05966A-s001

SC-015-D3SC05966A-s002

SC-015-D3SC05966A-s003
