# Supplementary material for: Photochemical H2 activation by an Zn–Fe heterometallic: a mechanistic investigation
Source: Chem Sci. 2023 Dec 14;15(4):1424–30. doi: 10.1039/d3sc05966a (PMC10806748; doi:10.1039/d3sc05966a)
Supplement: SC-015-D3SC05966A-s002 [file SC-015-D3SC05966A-s002.pdf]

Electronic Supplementary Material (ESI) for Chemical Science.
This journal is © The Royal Society of Chemistry 2023

Supporting Information

Photochemical H_2 Activation by a Zn–Fe Heterometallic: A Mechanistic Investigation

Marina Perez-Jimenez and Mark R. Crimmin*

Department of Chemistry, Molecular Sciences Research Hub, Imperial College London,
82 Wood Lane, White City, London, W12 0Z, UK.

1. Experimental section 1

2. NMR spectra 10

3. UV-Vis spectra 16

4. EPR experiments 17

5. Crystallographic data – X ray diffraction 20

6. DFT calculations 22

7. XYZ coordinates 27

8. References 37

1. Experimental section

All manipulations were carried out using standard Schlenk and glovebox techniques, under inert atmosphere (nitrogen or argon). A MBRAUN Labmaster glovebox was employed operating with concentrations of H_2O and O_2 below 0.1 ppm. Anhydrous solvents were obtained from a Grubbs type SPS system and stored over activated 3 Å molecular sieves under inert atmosphere. Glassware was dried for 12 hours at 120 °C prior to use. Benzene-d_6 was dried over 3 Å molecular sieves and degassed by freeze-pump-thaw cycles. Compounds **1a**,[1] **1b**[2] and **2a**[3] were prepared following the already reported literature procedures. [CpFe(CO)$_2$]$_2$ and [Cp'Fe(CO)$_2$]$_2$ (Cp = C_5H_5, Cp' = $C_5H_4(CH_3)$) were obtained from commercial suppliers (Sigma-Aldrich).

^{1}H, ^{2}H, ^{13}C{^{1}H} NMR spectra and two-dimensional experiments (e.g., COSY, HSQC, HMBC) were conducted in J. Young's NMR tubes on BRUKER 400 MHz or 500 MHz spectrometers. Chemical shifts (δ) were referenced to internal solvent resonances. Data was processed using the MestreNova and TopSpin software packages. NMR yields were calculated using benzene-d_6 solutions of ferrocene inserts and ^{1}H NMR experiments with d_1=5 acquisition parameters were used. Microanalysis (CHN) were performed under inert atmosphere by Elemental Microanalysis Ltd and London Metropolitan University. Infrared spectra were obtained on a Cary630 spectrometer (placed with in an MBraun glovebox) and UV-Vis spectra were collected in a Agilent Technologies Cary 60 UV-VIS. For the photochemical reactions, Kessil lamps (40 W, 428 nm and 390 nm) were used at 5 cm distance of the reaction vessels.

Single crystal X-ray data for compounds **2a**, **2b** and **3a** were collected using an Agilent Xcalibur PX Ultra A diffractometer, and the structures were refined using OLEX2 and SHELX-2019 program systems.[4] Details of the individual data collection are provided in the relevant CIFs. A summary of the fundamental crystal and refinement data are given in Table S1. Atomic coordinates, anisotropic displacement parameters and bond lengths and angles can be found in the .cif files, which have been deposited in the Cambridge Crystallographic Data Centre.

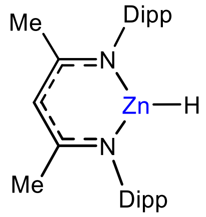

1a

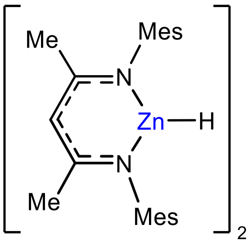

1b

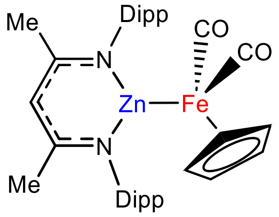

2a

[1] M. Garçon, N. W. Mun, A. J. P. White, M. R. Crimmin, *Angew. Chem. Int. Ed.* **2021**, *60*, 2–11.

[2] S. Schulza, T. Eisenmanna, D. Schuchmanna, M. Bolteb, M. Kirchnera, R. Boesea, J. Spielmanna, S. Harder, *Z. Naturforsch.* **2009**, *64b*, 1397–1400.

[3] T. J. Mazzacano, N. P. Mankad, *J. Am. Chem. Soc.* **2013**, *135*, 17258–17261.

[4] O. V. Dolomanov, L. J. Bourhis, R. J. Gildea, J. A. K. Howard, H. Puschmann, *J. Appl. Crystallogr*. **2009**, 42, 339–341; SHELX-2013, G.M. Sheldrick, *Acta Cryst*., **2015**, C71, 3–8.

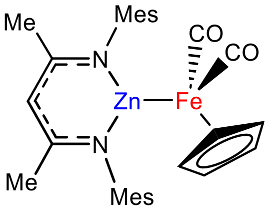

2b

NMR scale reaction of **1b** and $[CpFe(CO)_2]_2$: **1b** (10 mg, 0.013 mmol) and $[CpFe(CO)_2]_2$ (5 mg, 0.013 mmol) were dissolved in benzene-d_6 (0.5 mL) and the solution was transferred into a J. Young NMR tube. After 5 minutes at room temperature, the ^1H NMR spectrum showed clean formation of complex **2b** with elimination of H_2. Spectroscopic data match those obtained from an independent synthesis.

*Synthesis of **2b***: NaFp[5] (22 mg, 0.11 mmol) and of ^{Mes}(BDI)Zn–I[6] (50 mg, 0.11 mmol) were dissolved in THF (10 mL) and stirred for 1 hour at room temperature. The solvent was removed under vacuum and toluene (6 mL) was added. The solution was filtered through celite to give a yellow solution. After removing all the solvent under vacuum, *n*-pentane (4 mL) was added and the resulting insoluble solid was filtered and dried under reduced pressure. **2b** was obtained as a brown yellow solid (36 mg, 0.06 mmol, 56% yield). To obtain crystals suitable for X-ray diffraction studies, the brown yellow solid was dissolved in diethyl ether (4 mL) and the yellow solution was filtered and placed in the glovebox freezer at -35 °C for 2 days.

^1H NMR (400 MHz, C_6D_6, 298 K) δ (ppm): 1.64 (s, 6H, CMe), 2.17 (s, 6H, *p*-Me, Mes), 2.23 (s, 12H, *o*-Me, Mes), 3.93 (s, 5H, Cp), 5.02 (s, 1H, CH), 6.86 (s, 4H, *m*-CH, Mes).

$^{13}C\{^1H\}$ NMR (100 MHz, C_6D_6, 298 K) δ (ppm): 18.9 (Me, *o*-Mes), 21.0 (Me, *p*-Mes), 23.2 (C*Me*), 78.4 (Cp), 96.2 (CH), 129.6 (*m*-CH, Mes), 131.7 (*o*-C, Mes), 134.3 (*ipso*-C, Mes), 145.8 (*p*-C, Mes), 167.3 (*C*Me), 217.6 (CO).

IR: 1959, 1898 cm^{-1}

EA: Anal. Calcd. ($C_{30}H_{34}FeN_2O_2Zn$): C, 62.57; H, 5.95; N, 4.86. Found: C, 62.48; H, 5.99; N, 4.73.

---

[5] NaFp was prepared by reduction of Fp_2 following the literature procedures: M. E. Giuseppetti, A. R. Cutler, *Organometallics* **1987**, *6*, 970–973.

[6] ^{Mes}(BDI)Zn–I was prepared according to the literature procedures: S. Schulz, T. Eisenmann, U. Westphal, S. Schmidt, U. Flörke, *Z. Anorg. Allg. Chem*. **2009**, *635*, 216–220.

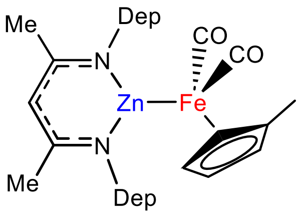

2c

*Synthesis of **2c***: NaFp’[7] (47 mg, 0.22 mmol) and of ^{Dep}(BDI)Zn–Cl[8] (100 mg, 0.22 mmol) were dissolved in THF (15 mL) and stirred for 1 hour at room temperature. The solvent was removed under vacuum and diethyl ether (10 mL) was added. The mixture was filtered through celite to give a yellow solution. After removing all the solvent under vacuum, the residue was washed with cold *n*-pentane (5 mL) and dried under reduced pressure. **2c** was obtained as an orange/yellow solid (80 mg, 0.13 mmol, 59% yield).

^{1}H NMR (400 MHz, C_6D_6, 298 K) δ (ppm): 1.24 (t, 12H, CH_3, Dep, $^3J_{HH}$=7.5 Hz), 1.48 (s, 3H, CH_3, Cp^{Me}), 1.66 (s, 6H, CMe), 2.62 (dq, 4H, CH_2, Dep, $^2J_{HH}$=15.1 Hz, $^3J_{HH}$=7.5 Hz), 2.77 (dq, 4H, CH_2, Dep, $^2J_{HH}$=15.1 Hz, $^3J_{HH}$=7.5 Hz), 3.68 (m, 2H, $(C_5H_4)^{Me}$), 3.94 (m, 2H, $(C_5H_4)^{Me}$), 5.02 (s, 1H, CH), 7.12 (s, 6H, *m*-CH and *p*-CH, Dep).

^{13}C{^{1}H} NMR (100 MHz, C_6D_6, 298 K) δ (ppm): 13.4 (CH_3, Cp^{Me}), 13.9 (CH_3, Dep), 23.6 (C*Me*), 25.0 (CH_2, Dep), 76.0 (CH, *Cp*^{Me}), 80.0 (CH, *Cp*^{Me}), 96.0 (CH), 97.9 (C, *Cp*^{Me}), 125.7 (*p*-CH, Dep), 126.3 (*m*-C, Dep), 137.3 (*o*-C, Dep), 147.4 (*ipso*-C, Dep), 167.2 (*C*Me), 218.2 (CO).

IR: 1952, 1889 cm^{-1}

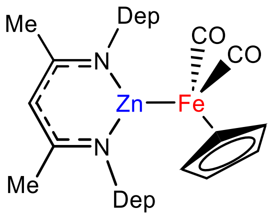

2e

*Synthesis of **2e***: NaFp (43 mg, 0.22 mmol) and of ^{Dep}(BDI)Zn–Cl (100 mg, 0.22 mmol) were dissolved in THF (10 mL) and stirred for 1 hour at room temperature. The solvent was removed under vacuum and toluene (6 mL) was added. The solution was filtered through celite to give an orange solution. After removing all the solvent under vacuum, the residue was washed with *n*-pentane (4 mL) and dried under reduced pressure. **2e** was obtained as an orange solid (70 mg, 0.12 mmol, 53% yield).

^{1}H NMR (400 MHz, C_6D_6, 298 K) δ (ppm): 1.23 (t, 12H, CH_3, Dep, $^3J_{HH}$=7.5 Hz), 1.66 (s, 6H, CMe), 2.60 (dq, 4H, CH_2, Dep, $^2J_{HH}$=15.1 Hz, $^3J_{HH}$=7.5 Hz), 2.75 (dq, 4H, CH_2, Dep, $^2J_{HH}$=15.1 Hz, $^3J_{HH}$=7.5 Hz), 3.88 (s, 5H, Cp), 5.02 (s, 1H, CH), 7.12 (m, 6H, *m*-CH and *p*-CH, Dep).

^{13}C{^{1}H} NMR (100 MHz, C_6D_6, 298 K) δ (ppm): 13.9 (CH_3, Dep), 23.6 (C*Me*), 25.0 (CH_2, Dep), 78.5 (Cp), 96.0 (CH), 125.8 (*p*-CH, Dep), 126.3 (*m*-C, Dep), 137.3 (*o*-C, Dep), 147.3 (*ipso*-C, Dep), 167.3 (*C*Me), 217.6 (CO).

IR: 1956, 1906 cm^{-1}

---

[7] NaFp’ was prepared by reduction Fp’$_2$ following the same method as for NaFp.

[8] ^{Dep}(BDI)Zn–Cl was prepared according to the literature procedures: M. Cheng, D. R. Moore, J. J. Reczek, B. M. Chamberlain, E. B. Lobkovsky, G. W. Coates, *J. Am. Chem. Soc.* **2001**, *123*, 8738–8749.

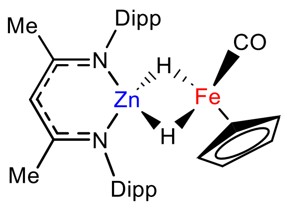

3a

NMR scale reaction of **1a** with $[CpFe(CO)_2]_2$: **1a** (10 mg, 0.02 mmol) and $[CpFe(CO)_2]_2$ (3.5 mg, 0.01 mmol) were dissolved in benzene-*d*$_6$ (0.5 mL) and transferred to a borosilicate J. Young NMR tube. The reaction mixture was exposed to light (LED Kessil lamp, 428 nm-40W) for a total of 6 hours and the transformation was monitored by ^{1}H NMR spectroscopy leading to a 1:1 mixture of **2a** and **3a** (65% NMR yield). Crystals suitable for X-ray diffraction studies were obtained from this reaction. The solvent was removed under vacuum inside the glovebox and diethyl ether (1 mL) was added, the solution was filtered and placed in the freezer for 2 days at -35 °C. The structure of complex **3a** co-crystallizes with one molecule of the Zn−Fe adduct (**2a**).

NMR scale reaction of **1a** with $[CpFe(CO)_2]_2$ in the presence of H_2: **1a** (10 mg, 0.02 mmol) and $[CpFe(CO)_2]_2$ (3.5 mg, 0.01 mmol) were dissolved in benzene-*d*$_6$ (0.5 mL) and transferred to a borosilicate J. Young NMR tube. The tube was then freeze-pump thawed and the N_2 atmosphere was replaced by H_2 atmosphere (1 bar). Then, the tube was exposed to light (LED Kessil lamp, 390 nm-40W) for a total of 8 hours and the transformation was monitored by ^{1}H NMR spectroscopy (Figure S1) leading to a 0.2:0.8 mixture of **2a** and **3a** (97% NMR yield).

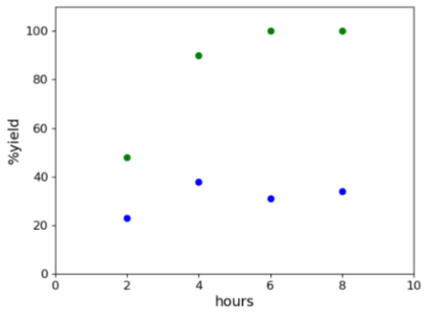

Figure S1. Formation of **3a** (green dots) and **2a** (blue dots) over time.

NMR scale reaction of **2a** with H_2: **2a** (10 mg, 0.016 mmol) were dissolved in benzene-*d*$_6$ (0.5 mL) and transferred to a borosilicate J. Young NMR tube. The tube was then freeze-pump thawed and the N_2 atmosphere was replaced by H_2 atmosphere (1 bar). Then, the tube was exposed to light (LED Kessil lamp, 428 nm-40W) for a total of 6 hours and the transformation was monitored by ^{1}H NMR leading to a mixture of **3a**, $[CpFe(CO)_2]_2$ and **1a** in 52%, 10% and 15% NMR yields, respectively. When the tube was exposed to a different light source (LED Kessil lamp 390 nm-40W) for 6 hours, the NMR yields were: 55%, 26% and 20 % for **3a**, $[CpFe(CO)_2]_2$ and **1a** respectively.[9]

*Synthesis of **3a***: **2a** (50 mg, 0.08 mmol) was dissolved in C_6H_6 (5 mL) and the yellow solution was transferred into an ampoule. The ampoule was freeze-pump thawed and the atmosphere was changed to dihydrogen (1 bar). The mixture was stirred for 6 hours in the presence of blue LED light (390 nm-40 W). After removing all the solvent under vacuum, diethyl ether (4 mL) was added forming an orange solution which was filtered forming an orange/yellow solid after removal of the solvent (21 mg, 0.04 mmol, 45% yield).

[9] Higher pressure of H_2 were also employed (4 bar) and although lower yields for the by-products Fp_2 and **1a** were obtained (<10 %), lower conversions were observed and the yield for **3a** could not be improved.

^{1}H NMR (400 MHz, C_6D_6, 298 K) δ (ppm): -15.98 (s, 2H, Zn(H)$_2$Fe), 1.14 (d, 12H, CH(C*H*$_3$)$_2$, $^3J_{HH}$ = 6.7 Hz), 1.43 (d, 12H, CH(C*H*$_3$)$_2$, $^3J_{HH}$ = 6.7 Hz), 1.72 (s, 6H, CCH$_3$), 3.25 (sept, 4H, C*H*(CH$_3$)$_2$, $^3J_{HH}$ = 6.7 Hz), 3.79 (s, 5H, Cp), 4.97 (s, 1H, CH), 7.13 (m, 6H, *m*-CH, *p*-CH, Dipp).

^{13}C{^{1}H} NMR (100 MHz, C_6D_6, 298 K) δ (ppm): 23.8 (C*Me*), 24.3 (CH*Me*$_2$), 24.4 (CH*Me*$_2$), 28.5 (*C*HMe$_2$), 76.5 (Cp), 95.2 (CH), 124.2 (*m*-CH, Dipp), 126.3 (*p*-CH, Dipp), 142.1 (*i*-C, Dipp), 144.7 (*o*-C, Dipp), 167.7 (*C*Me), 218.1 (CO).

IR: 1780, 1810 (shoulder), 1948 (CO) cm^{-1}

EA: Anal. Calcd. ($C_{35}H_{48}FeN_2OZn$): C, 66.31; H, 7.63; N, 4.42. Found: C, 63.77; H, 6.74; N, 4.00. The CHN analysis results do not match well those predicted, likely due to decomposition and possibly loss of CO.

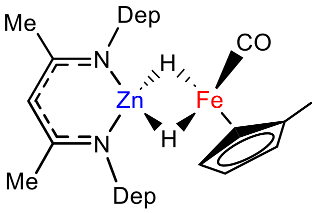

3c

NMR scale reaction of **2c** with H_2: **2c** (10 mg, 0.016 mmol) were dissolved in benzene-*d*$_6$ (0.5 mL) and transferred to a borosilicate J. Young NMR tube. The tube was then freeze-pump thawed and the N_2 atmosphere was replaced by H_2 atmosphere (1 bar). Then, the tube was exposed to light (LED Kessil lamp, 390 nm-40W) for a total of 6 hours and the transformation was monitored by ^{1}H NMR leading to formation of **3c** in 43% NMR yield. The yellow solution in benzene was transferred to a vial inside the glovebox and the solvent was removed under vacuum, the residue was washed with cold *n*-pentane (1 mL) leading to a yellow solid (4 mg, 0.006 mmol, 36 % yield).

^{1}H NMR (400 MHz, C_6D_6, 298 K) δ (ppm): -15.90 (s, 2H, Zn(H)$_2$Fe), 1.26 (t, 12H, CH$_3$, Dep, $^3J_{HH}$=7.5 Hz), 1.57 (s, 3H, CH$_3$, Cp^{Me}), 1.63 (s, 6H, CMe), 2.57 (dq, 4H, CH$_2$, Dep, $^2J_{HH}$=15.1 Hz, $^3J_{HH}$=7.5 Hz), 2.74 (dq, 4H, CH$_2$, Dep, $^2J_{HH}$=15.1 Hz, $^3J_{HH}$=7.5 Hz), 3.59 (m, 2H, (C_5H_4)^{Me}), 3.73 (m, 2H, (C_5H_4)^{Me}), 4.93 (s, 1H, CH), 7.11 (m, 6H, *m*-CH and *p*-CH, Dep).

^{13}C{^{1}H} NMR (100 MHz, C_6D_6, 298 K) δ (ppm): 13.9 (CH$_3$, Cp^{Me}), 14.1 (CH$_3$, Dep), 23.4 (C*Me*), 24.9 (CH$_2$, Dep), 75.3 (CH, *Cp*^{Me}), 75.9 (CH, Cp^{Me}), 95.3 (CH), 96.0 (C, *Cp*^{Me}), 125.7 (*p*-CH, Dep), 126.4 (*m*-C, Dep), 137.3 (*o*-C, Dep), 146.7 (*ipso*-C, Dep), 167.3 (*C*Me), 218.2 (CO).

IR: 1773, 1942 (CO) cm^{-1}

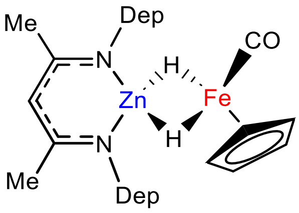

3e

NMR scale reaction of **2e** with H_2: **2e** (10 mg, 0.016 mmol) were dissolved in benzene-*d*$_6$ (0.5 mL) and transferred to a borosilicate J. Young NMR tube. The tube was then freeze-pump thawed and the N_2 atmosphere was replaced by H_2 atmosphere (1 bar). Then, the tube was exposed to light (LED Kessil

lamp, 390 nm-40W) for a total of 6 hours and the transformation was monitored by ^{1}H NMR leading to formation of **3e** in 40% NMR yield.

^{1}H NMR (400 MHz, C_6D_6, 298 K) δ (ppm): -15.96 (s, 2H, $Zn(H)_2Fe$), 1.25 (br m, 12H, CH_3, Dep), 1.63 (s, 6H, CMe), 2.59 (br m, 4H, CH_2, Dep), 2.73 (br m, 4H, CH_2, Dep), 3.78 (s, 5H, Cp), 4.93 (s, 1H, CH), 7.09 (m, 6H, *m*-CH and *p*-CH, Dep).

Cross-over experiments:

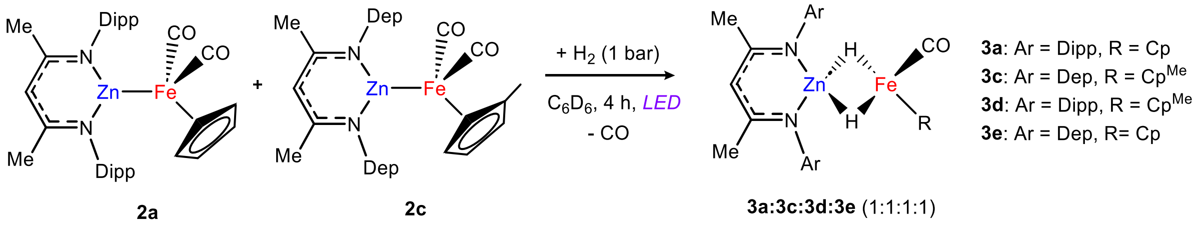

A) NMR scale reaction of **2a** and **2c** with H_2: **2a** (10 mg, 0.016 mmol) and **2c** (10 mg, 0.016 mmol) were dissolved in benzene-d_6 (0.5 mL) and transferred to a borosilicate J. Young NMR tube. The tube was then freeze-pump thawed and the N_2 atmosphere was replaced by H_2 atmosphere (1 bar). Then, the tube was exposed to light (LED Kessil lamp, 390 nm-40W) for a total of 4 hours and the transformation was monitored by ^{1}H NMR leading to a 1:1:1:1 mixture of **3a:3c:3d:3e** in 30% NMR yield.

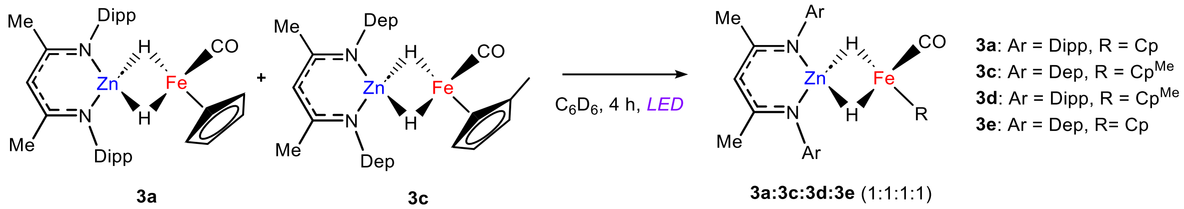

B) NMR scale reaction of **3a** and **3c**: **3a** (4 mg, 0.006 mmol) and **3c** (4 mg, 0.006 mmol) were dissolved in benzene-d_6 (0.5 mL) and transferred to a borosilicate J. Young NMR tube. The tube was exposed to light (LED Kessil lamp, 390 nm-40W) for a total of 4 hours and the transformation was monitored by ^{1}H NMR leading to the formation of **3d** and **3e** in 21 and 22% NMR yields.

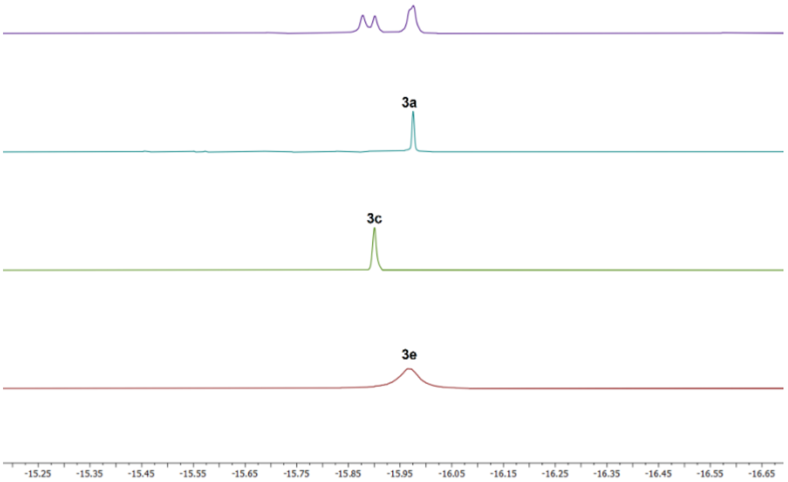

Figure S2. ^{1}H NMR spectra, hydride region fragment. From top to bottom: mixture of the four hydrides obtained via experiment **A**; independently synthesised hydrides species **3a**, **3c** and **3e**.

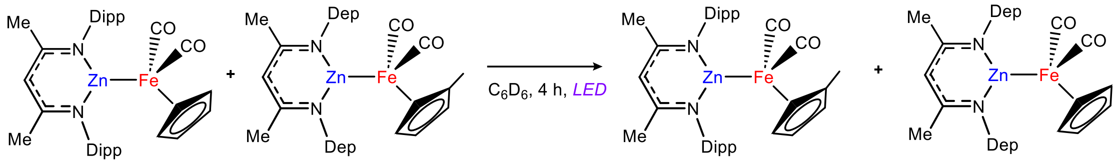

2a **2c** **2d** **2e**

C) NMR scale reaction of **2a** and **2c** in the absence of H_2: **2a** (10 mg, 0.016 mmol) and **2c** (10 mg, 0.016 mmol) were dissolved in benzene-d_6 (0.5 mL) and transferred to a borosilicate J. Young NMR tube. The tube was exposed to light (LED Kessil lamp, 390 nm-40W) for a total of 4 hours and the transformation was monitored by ^{1}H NMR leading to the formation of adducts **2d** and **2e** in 15% and 15% NMR yield, respectively. Iron dimers Fp, Fp'$_2$ and Fp–Fp' were observed as by-products in 3, 3 and 6% NMR yields, respectively.

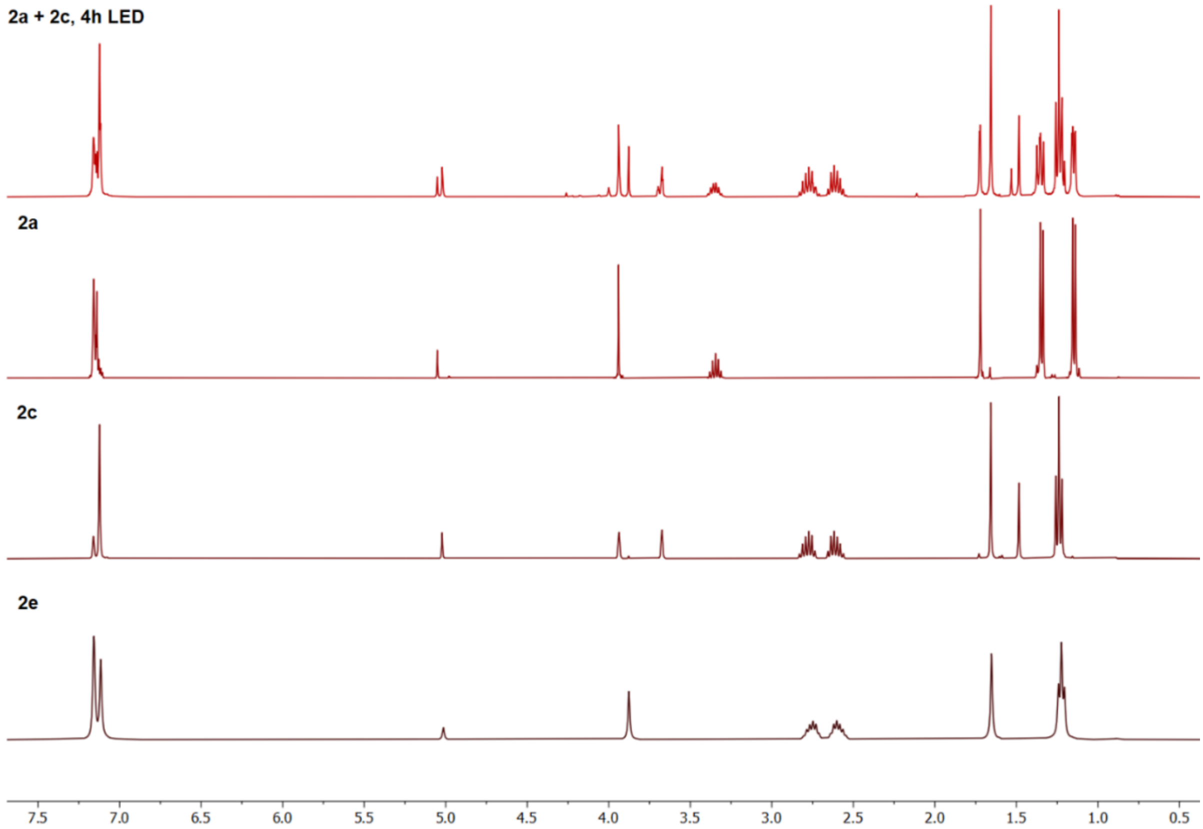

Figure S3 ^{1}H NMR spectra. From top to bottom: equimolar mixture of **2a** and **2c** after 4 h exposure to LED lamp (experiment **C**); independently synthesised adducts **2a**, **2c** and **2e**.

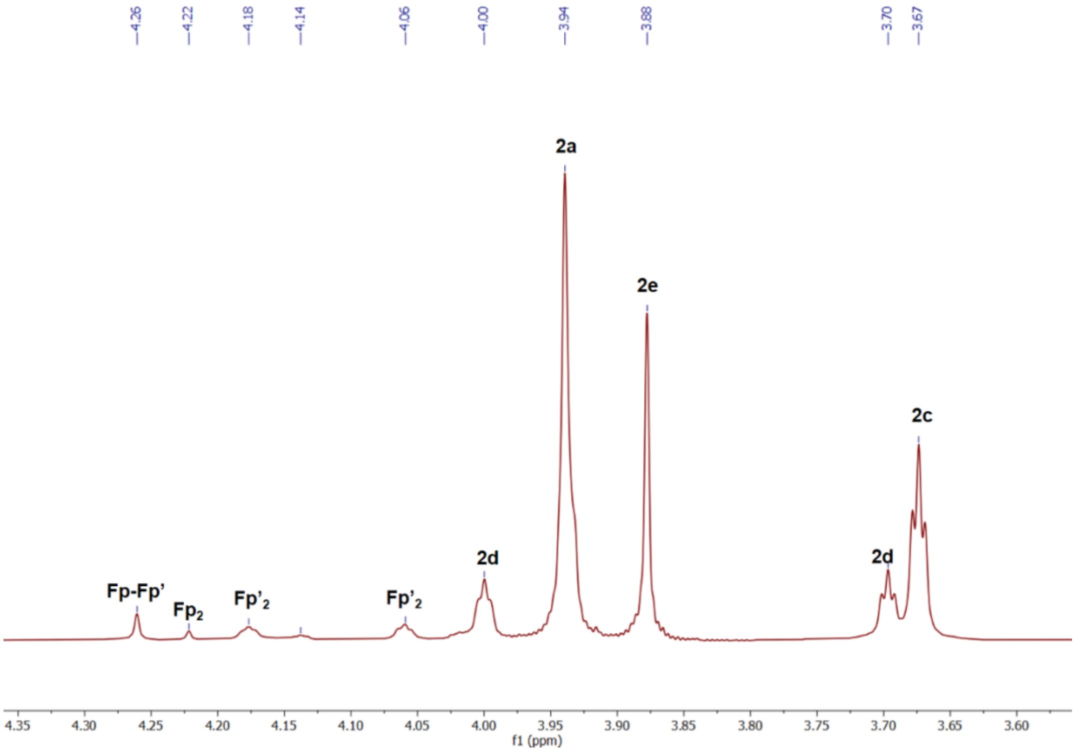

Figure S4. ^{1}H NMR spectrum fragment of equimolar mixture of **2a** and **2c** after 4 h exposure to LED lamp (experiment **C**).

Other experiments:

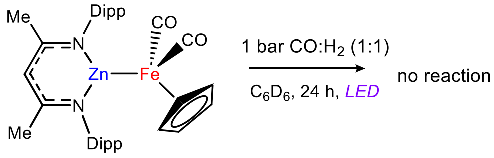

2a

NMR scale reaction of **2a** and syngas CO:H_2 (1:1 mixture): **2a** (10 mg, 0.016 mmol) were dissolved in benzene-d_6 (0.5 mL) and transferred to a borosilicate J. Young NMR tube. The tube was then freeze-pump thawed and the N_2 atmosphere was replaced by the syngas atmosphere (1 bar). The tube was exposed to light (LED Kessil lamp, 390 nm-40W) for a total of 24 hours and the transformation was monitored by ^{1}H NMR. Product **3a** was not observed.

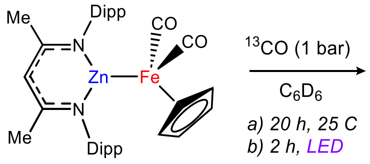

2a

NMR scale reaction of **2a** and ^{13}CO: **2a** (10 mg, 0.016 mmol) were dissolved in benzene-d_6 (0.5 mL) and transferred to a borosilicate J. Young NMR tube (2 parallel tubes). Both tubes were then freeze-pump thawed and the N_2 atmosphere was replaced by the ^{13}CO atmosphere (1 bar). Tube *a* was left at room temperature over 20 hours, while tube *b* was exposed to light (LED Kessil lamp, 390 nm-40W) for 2 hours and the transformation was monitored by ^{13}C NMR. The incorporation of ^{13}CO in the adduct was 40% higher in the photochemical reaction compared to the thermal conditions.

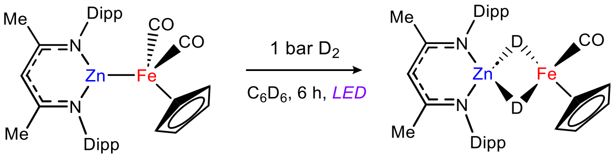

2a **3a-*d***

NMR scale reaction of **2a** and D_2: **2a** (10 mg, 0.016 mmol) were dissolved in benzene (0.5 mL) and transferred to a borosilicate J. Young NMR tube with a ferrocene capillary in tol-d_8. The tube was then freeze-pump thawed and the N_2 atmosphere was replaced by the D_2 atmosphere (1 bar). The tube was exposed to light (LED Kessil lamp, 390 nm-40W) for 6 hours and the transformation was monitored by ^{2}H NMR and ^{1}H NMR experiments. A 92% incorporation of deuterium was obtained for the final product **3a-*d***.

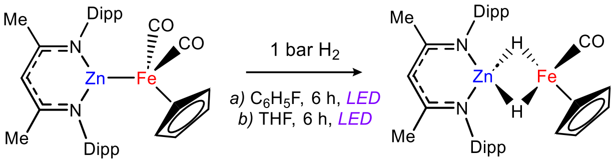

2a **3a**

Solvent screening:

a) **2a** (10 mg, 0.016 mmol) were dissolved in fluorobenzene (0.5 mL) and transferred to a borosilicate J. Young NMR tube. The tube was then freeze-pump thawed and the N_2 atmosphere was replaced by the H_2 atmosphere (1 bar). The tube was exposed to light (LED Kessil lamp, 390 nm-40W) for 6 hours and the transformation was monitored by ^{1}H NMR experiments leading to the formation of **3a** in a 47% NMR yield.

b) **2a** (10 mg, 0.016 mmol) were dissolved in THF (0.5 mL) and transferred to a borosilicate J. Young NMR tube. The tube was then freeze-pump thawed and the N_2 atmosphere was replaced by the H_2 atmosphere (1 bar). The tube was exposed to light (LED Kessil lamp, 390 nm-40W) for 6 hours and the transformation was monitored by ^{1}H NMR experiments leading to the formation of **3a** in a 43% NMR yield.

2. NMR spectra

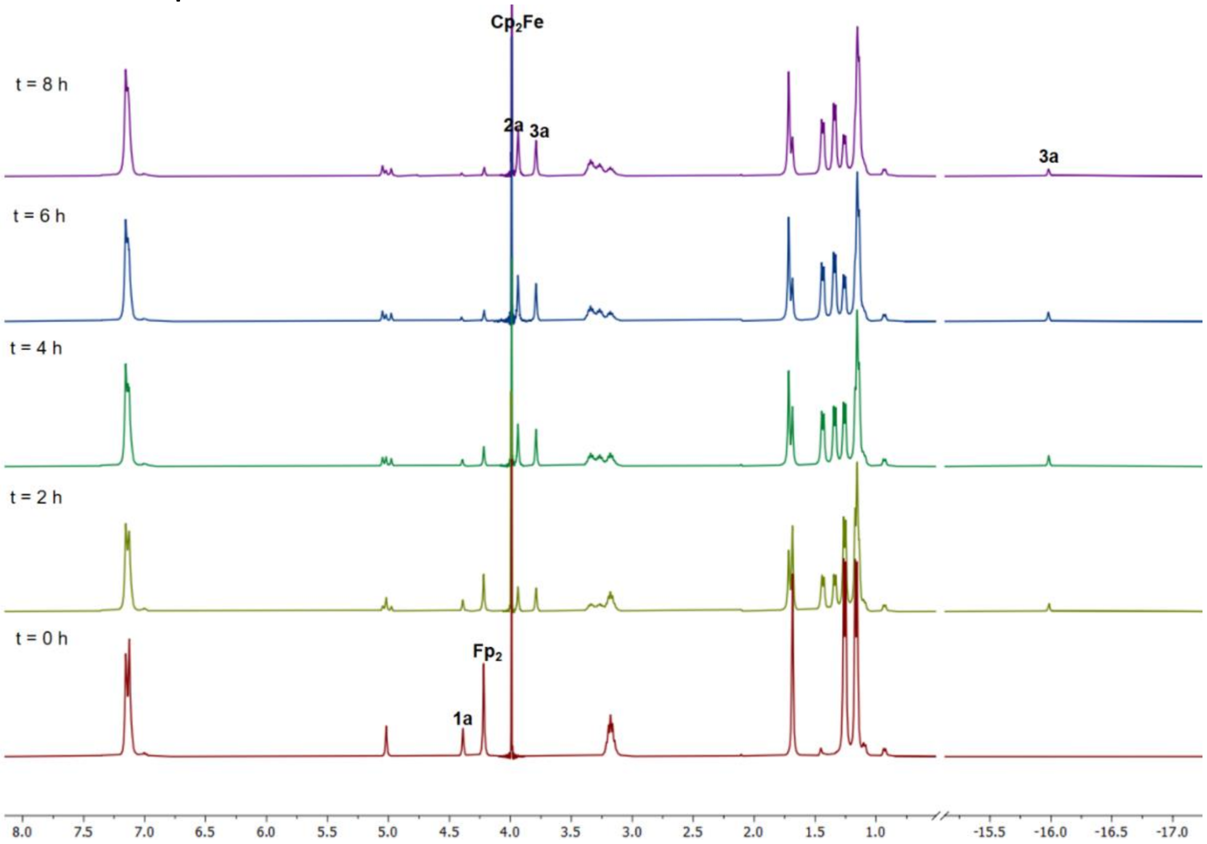

Figure S5. ^{1}H NMR (400 MHz, C_6D_6, 298K) monitoring of the reaction of **1a** with $[CpFe(CO)_2]_2$ under photochemical conditions (LED lamp, 428nm-40W).

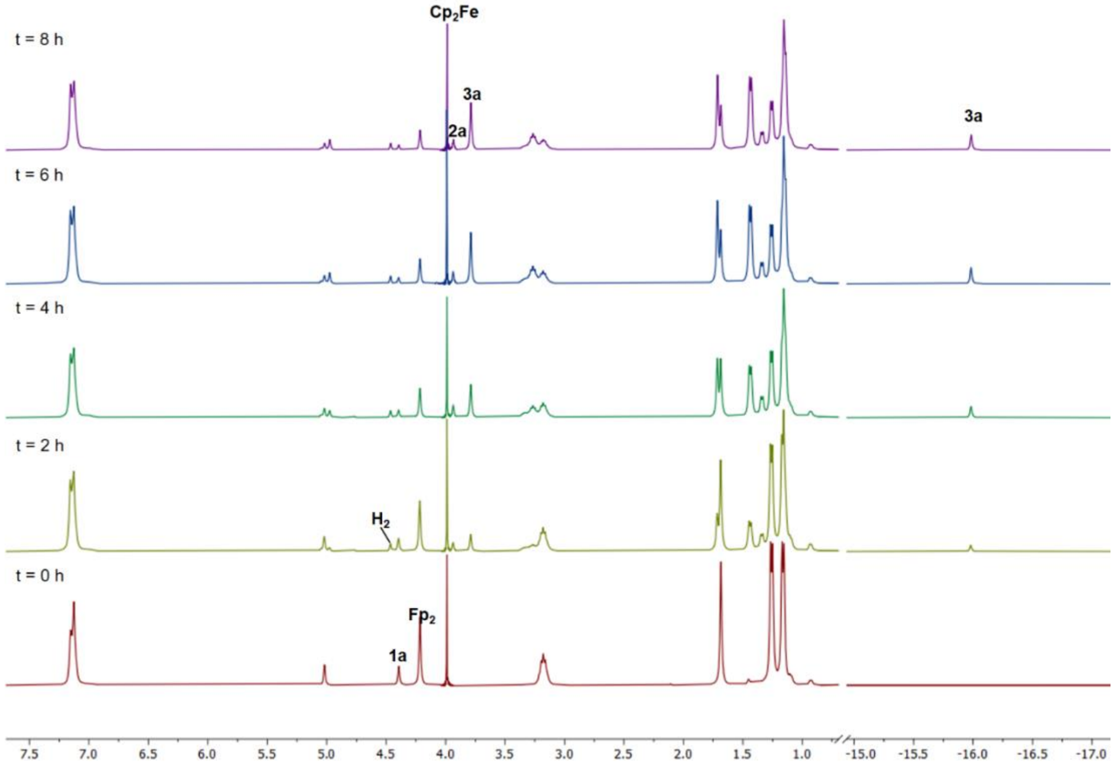

Figure S6. ^{1}H NMR (400 MHz, C_6D_6, 298K) monitoring of the reaction of **1a** with $[CpFe(CO)_2]_2$ in the

presence of H_2 (1 bar) under photochemical conditions (LED lamp, 390nm-40W).

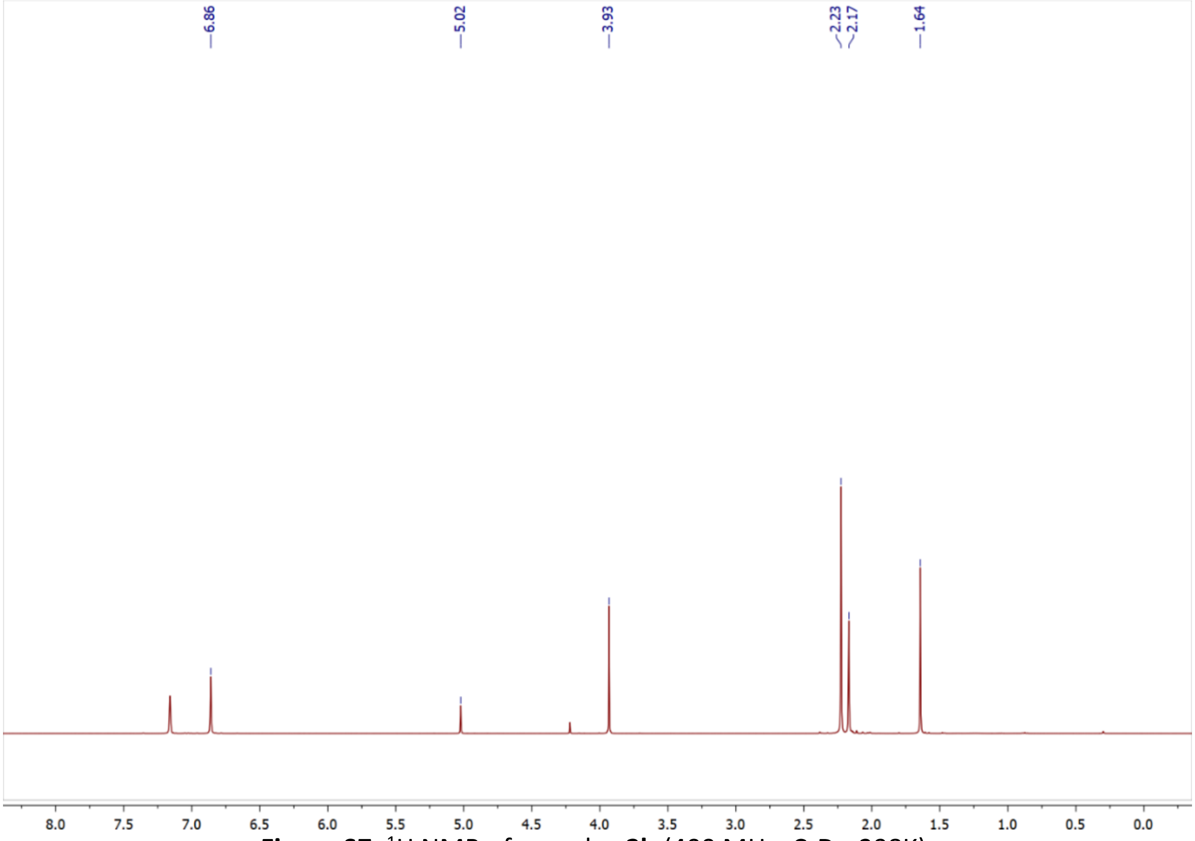

Figure S7. ^1H NMR of complex **2b** (400 MHz, C_6D_6, 298K).

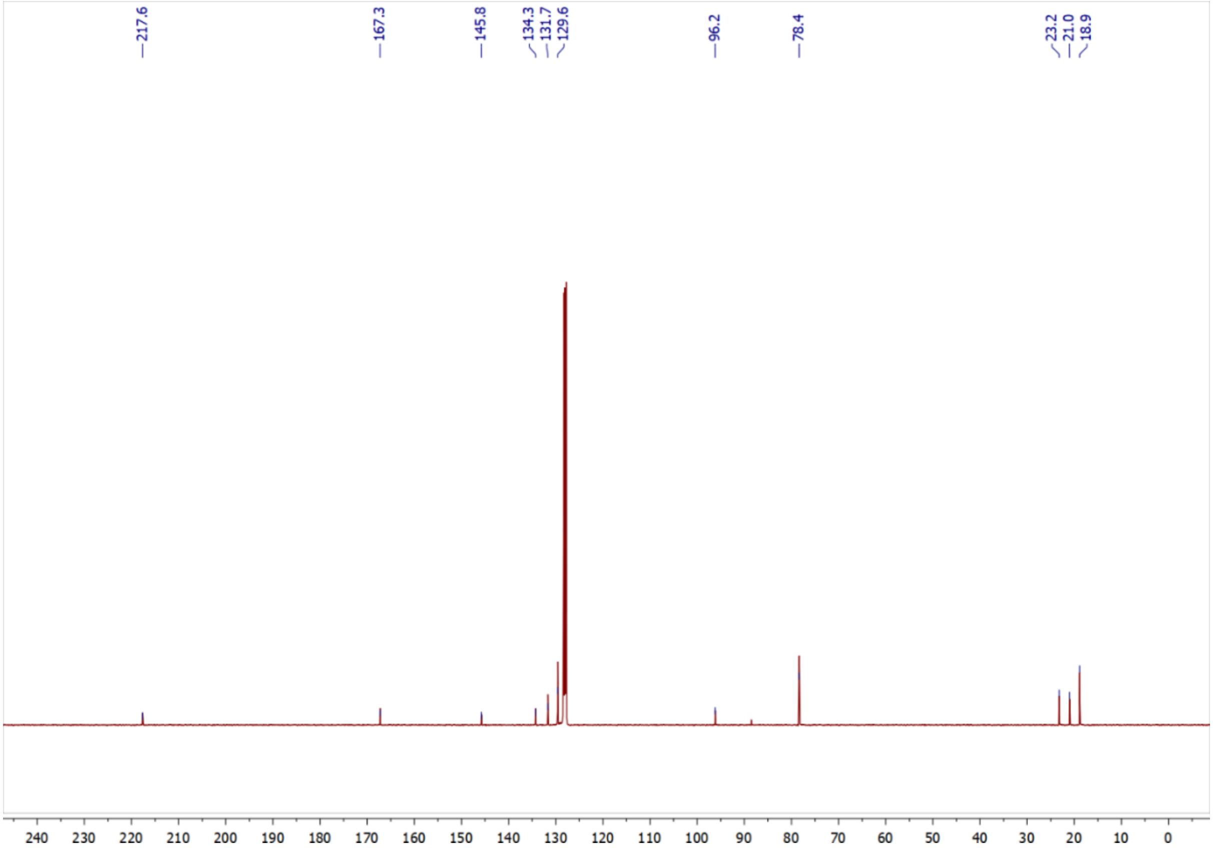

Figure S8. $^{13}C\{^{1}H\}$ NMR of complex **2b** (100 MHz, C_6D_6, 298K).

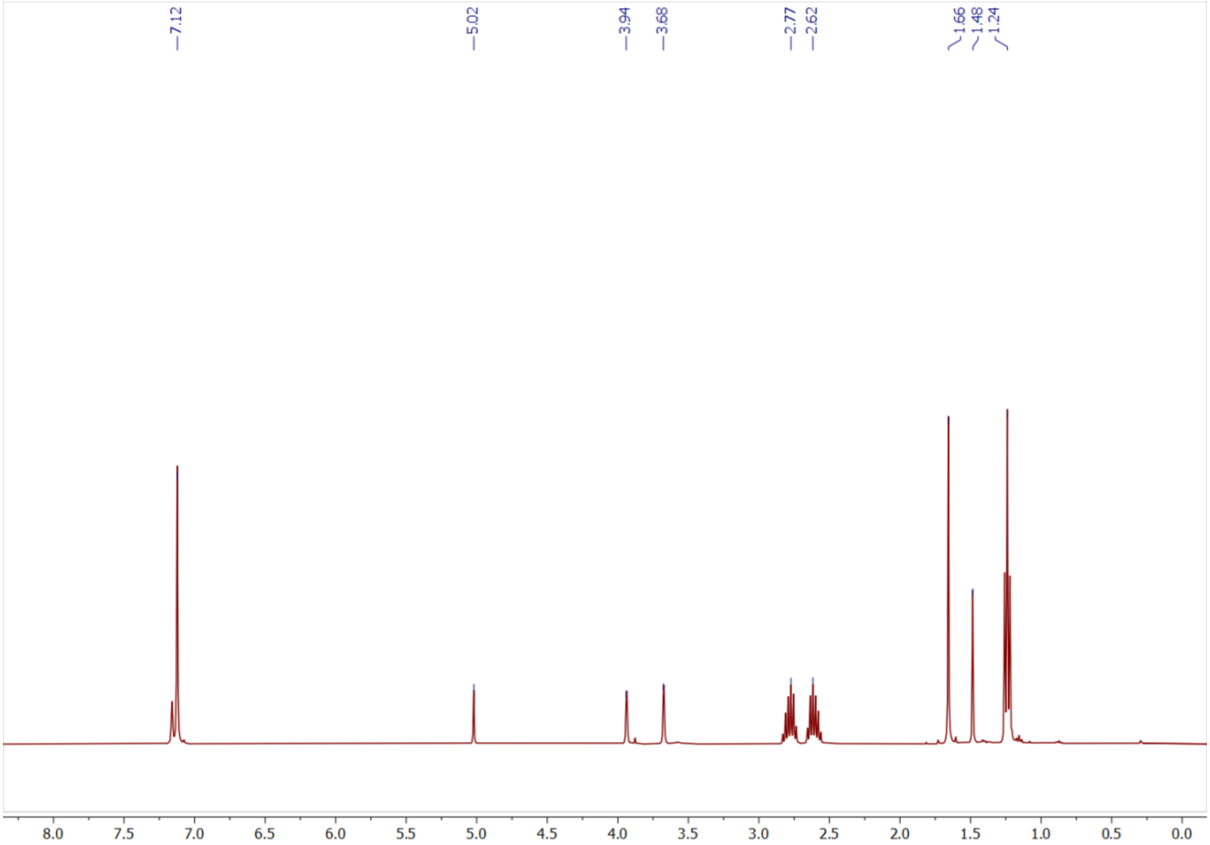

Figure S9. ^{1}H NMR of complex **2c** (400 MHz, C_6D_6, 298K).

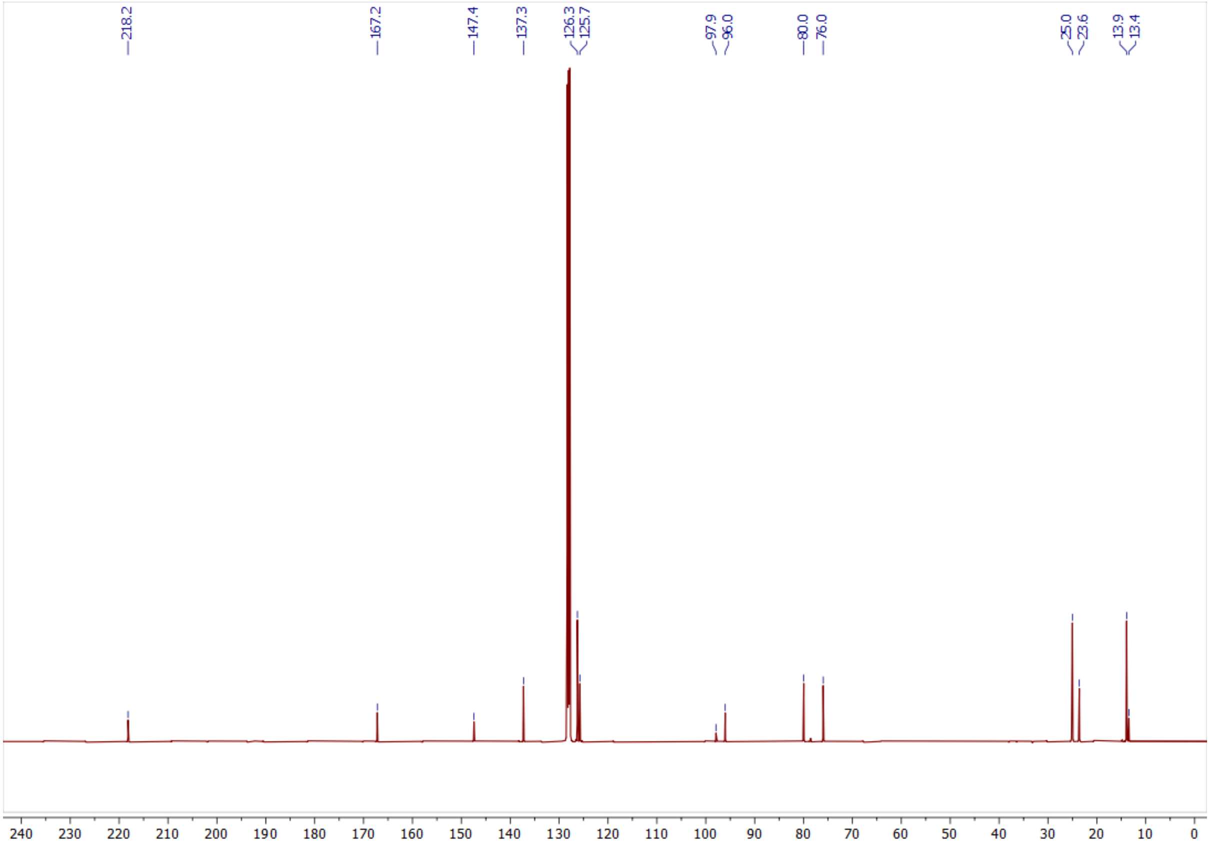

Figure S10. $^{13}C\{^1H\}$ NMR of complex **2c** (100 MHz, C_6D_6, 298K).

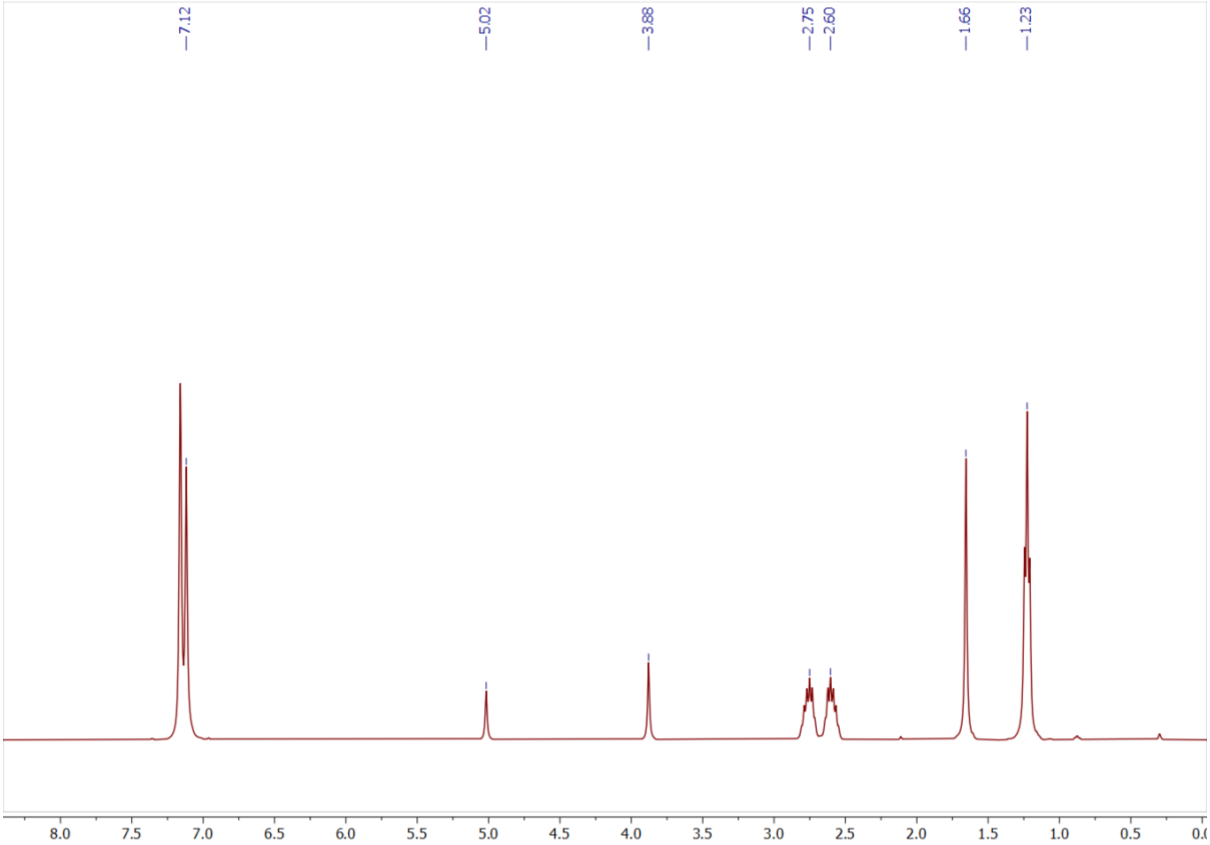

Figure S11. ^1H NMR of complex **2e** (400 MHz, C_6D_6, 298K).

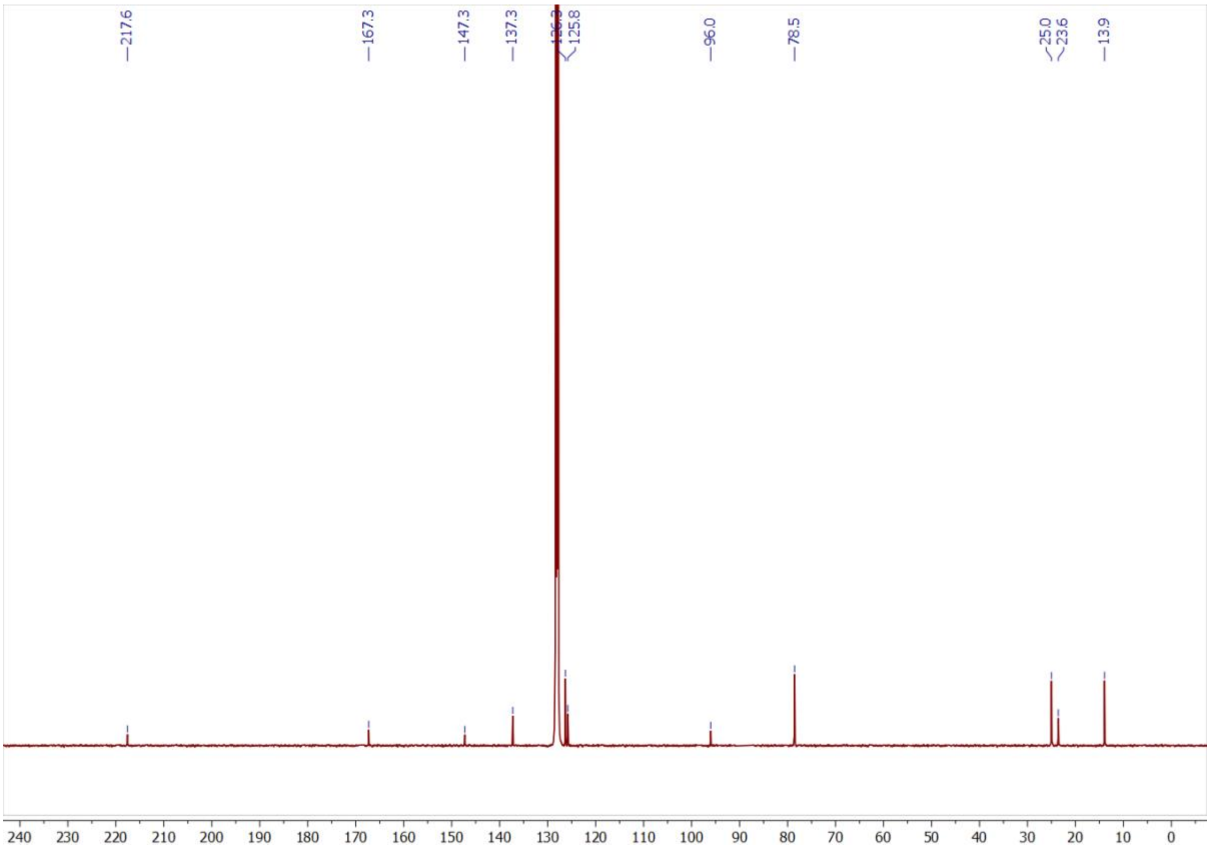

Figure S12. $^{13}C\{^1H\}$ NMR of complex **2e** (100 MHz, C_6D_6, 298K).

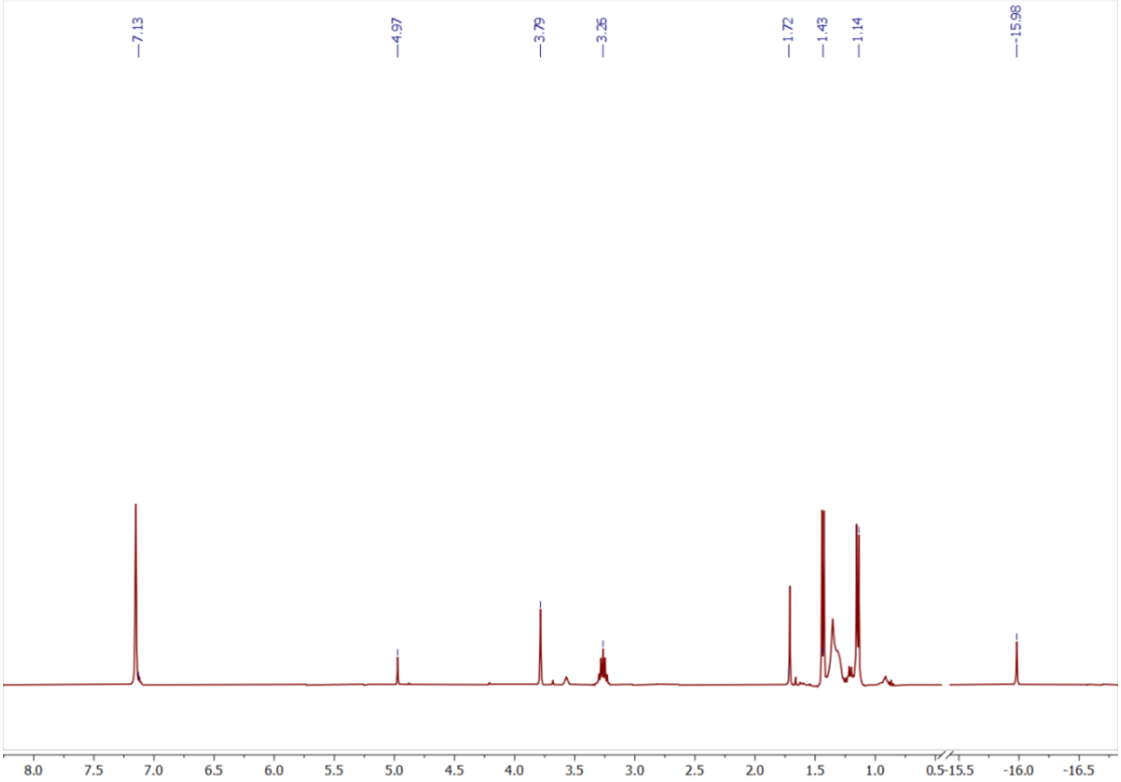

Figure S13. ^1H NMR of complex **3a** (400 MHz, C_6D_6, 298K).

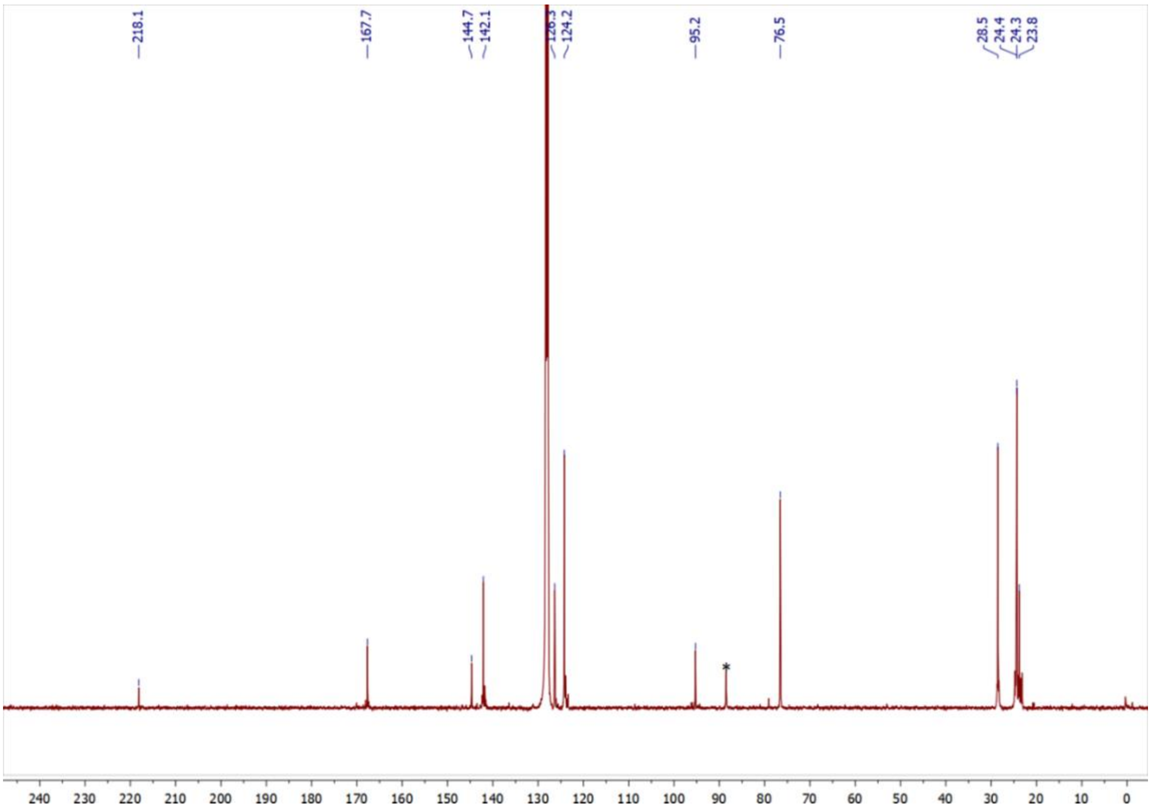

Figure S14. $^{13}C\{^1H\}$ NMR of complex **3a** (100 MHz, C_6D_6, 298K).*$[CpFe(CO)_2]_2$

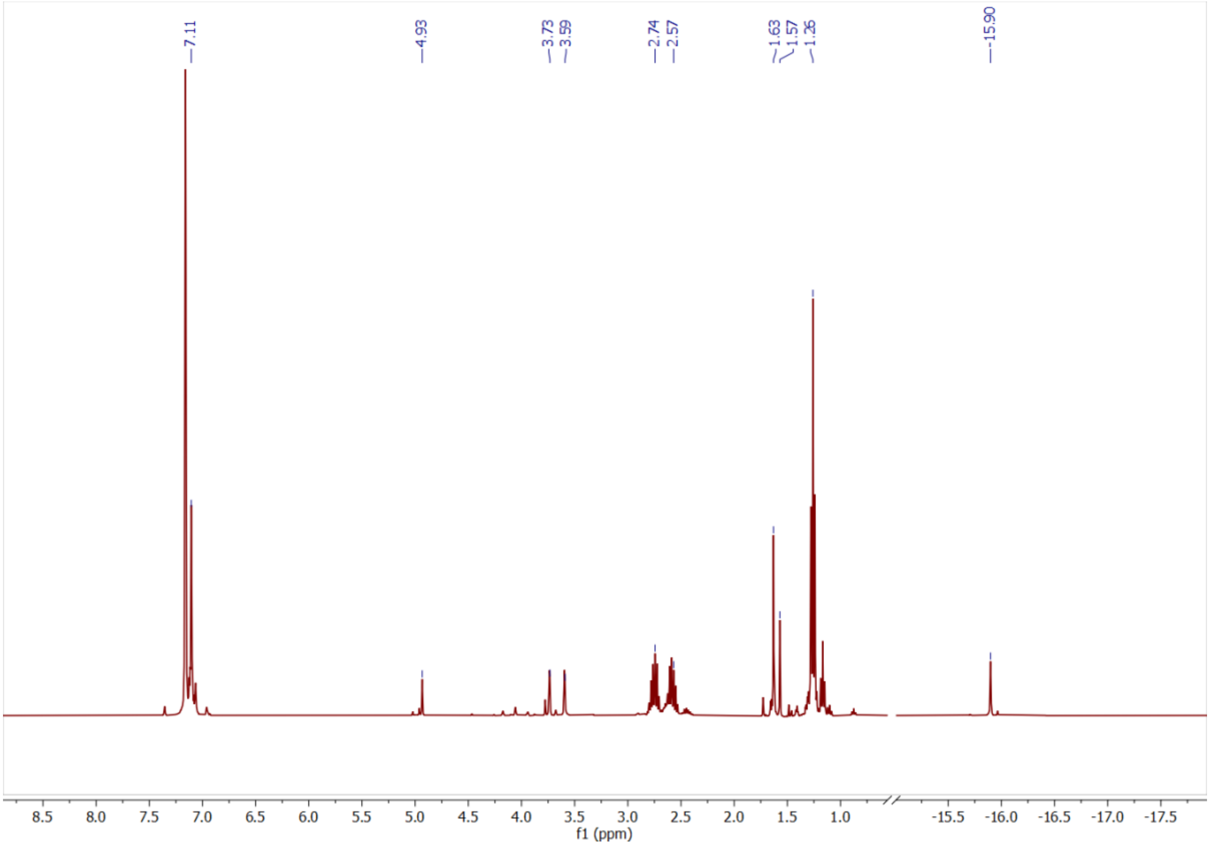

Figure S15. ^{1}H NMR of complex **3c** (400 MHz, C_6D_6, 298K).

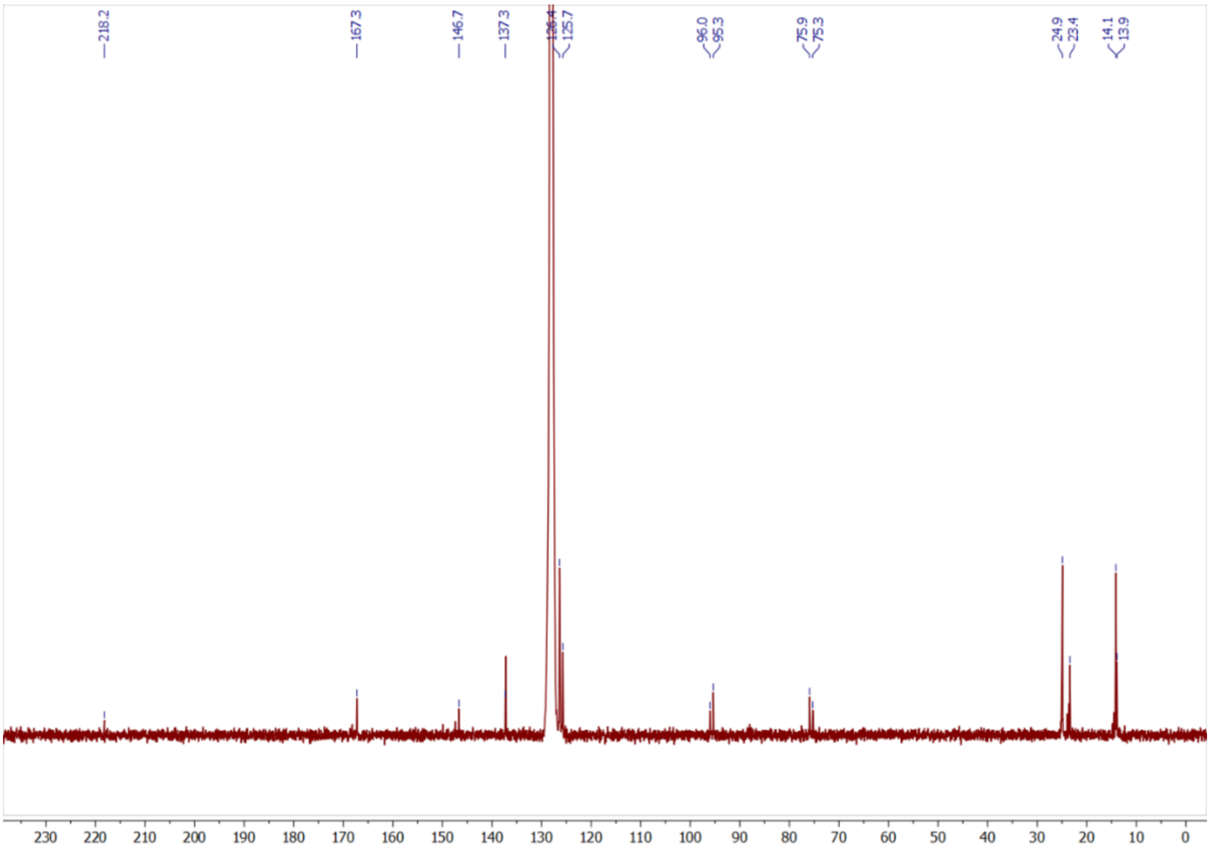

Figure S16. $^{13}C\{^{1}H\}$ NMR of complex **3c** (100 MHz, C_6D_6, 298K).

3. UV-VIS spectra

All of the UV-Vis spectra were collected in a Agilent Technologies Cary 60 UV-VIS. The samples were prepared inside the glovebox and placed in a Quartz cuvette with a J. Young tap of 4 mL volume. All of spectra were recorded in THF for 0.1 mM solutions of complexes **2a**, **2b**, **2c**, **3a** and **3c**.

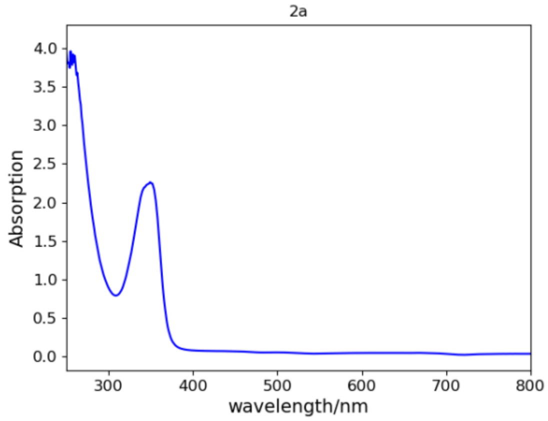

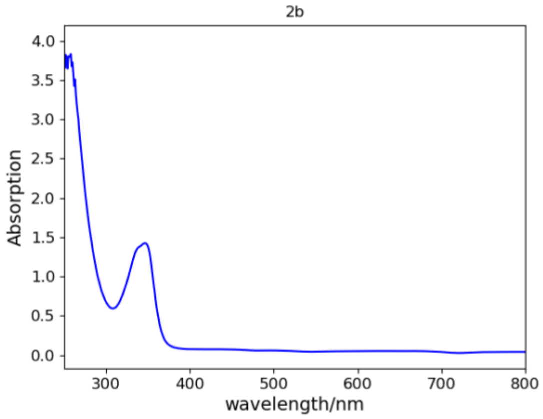

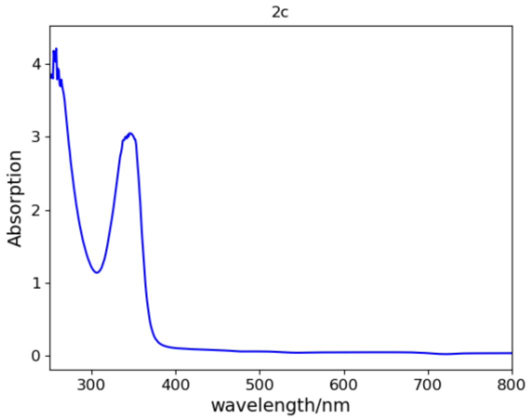

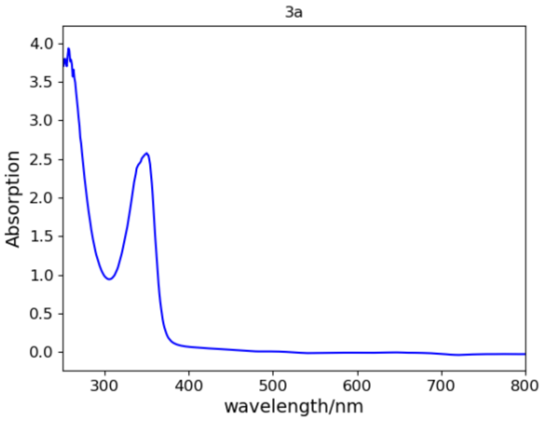

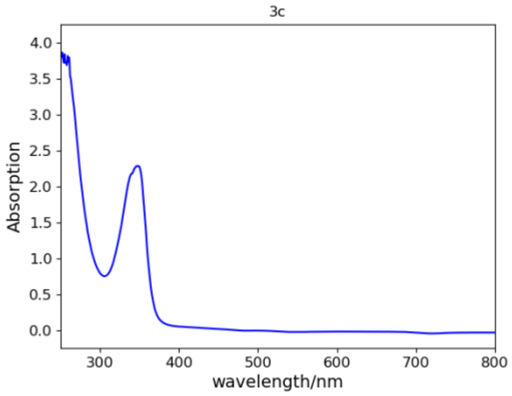

4. EPR experiments

Equipment details: The EPR experiments were performed in the PEPR-Centre for Pulse EPR Spectroscopy at Imperial College London in a benchtop CW X-band spectrometer (Magnettech ESR5000) at room temperature in benzene-d_6. The EPR experiments were recorded in 5-10 minutes time after the exposure to the LED light, whereas the ^{1}H NMR were recorded within 1 hour at room temperature after the exposure time.

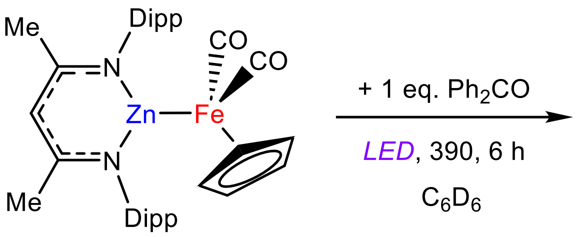

2a

NMR scale reaction of **2a** and 1 equivalent of benzophenone in the absence of H_2: **2a** (10 mg, 0.016 mmol) and benzophenone (3 mg, 0.016 mmol) were dissolved in benzene-d_6 (0.5 mL) and transferred to a borosilicate J. Young NMR tube. The tube was exposed to light (LED Kessil lamp, 390 nm-40W) for a total of 6 hours and the transformation was monitored by ^{1}H NMR and EPR experiments (Figure 4b, *main text*).

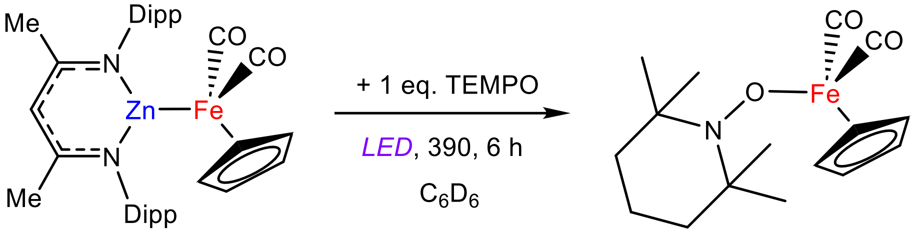

2a

NMR scale reaction of **2a** and 1 equivalent of TEMPO in the absence of H_2: **2a** (10 mg, 0.016 mmol) and TEMPO (2.5 mg, 0.016 mmol) were dissolved in benzene-d_6 (0.5 mL) and transferred to a borosilicate J. Young NMR tube. The tube was exposed to light (LED Kessil lamp, 390 nm-40W) for a total of 6 hours and the transformation was monitored by ^{1}H NMR showing [Fe(η^5-C_5H_5)(CO)$_2$(TEMPO)] (50 % NMR yield) and EPR experiments (Figure S17). The formation of [Fe(η^5-C_5H_5)(CO)$_2$(TEMPO)] was confirmed by addition of TEMPO to [Fe(η^5-C_5H_5)(CO)$_2$]$_2$ under the same photochemical conditions.

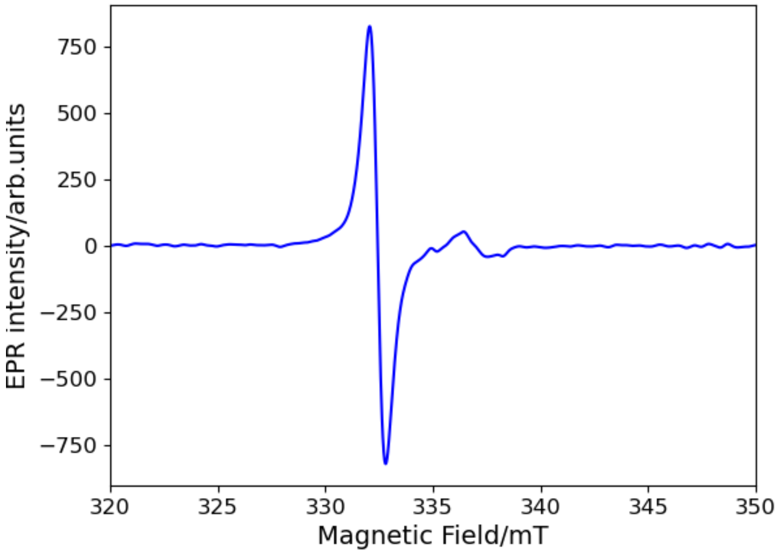

Figure S17. CW X-band EPR spectrum recorded in benzene solution at ambient temperature; microwave frequency 9.44769 GHz; field modulation amplitude, 0.25 mT.

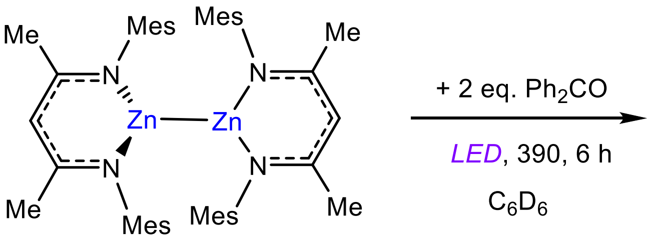

NMR scale reaction of [^{Mes}(BDI)Zn]$_2$ and 2 equivalents of benzophenone: [^{Mes}(BDI)Zn]$_2$ (10 mg, 0.012 mmol) and benzophenone (4 mg, 0.024 mmol) were dissolved in benzene-d_6 (0.5 mL) and transferred to a borosilicate J. Young NMR tube. The tube was exposed to light (LED Kessil lamp, 390 nm-40W) for a total of 6 hours and the transformation was monitored by ^{1}H NMR (46% NMR conversion) and EPR experiments (Figure S18).

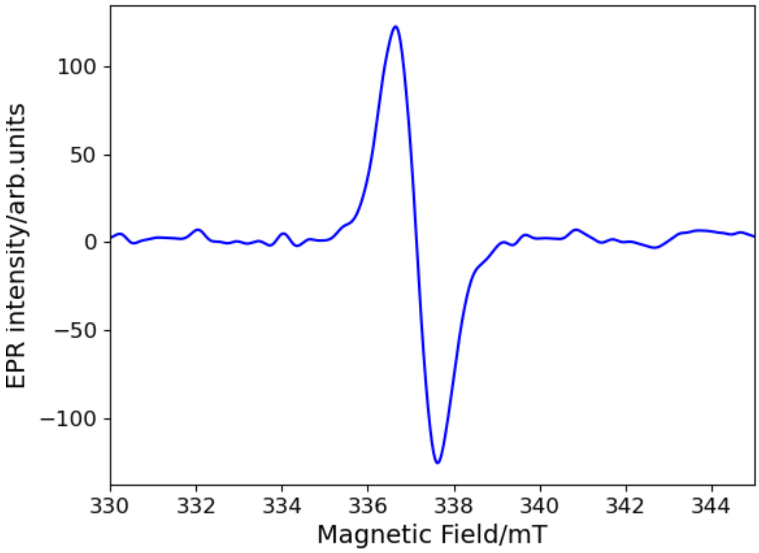

Figure S18. CW X-band EPR spectrum recorded in benzene solution at ambient temperature; microwave frequency 9.44728 GHz; field modulation amplitude, 0.25 mT.

5. Crystallographic data – X ray diffraction

	**2a + 3a**	**2b**
Formula	$C_{71}H_{94}Fe_2N_4O_3Zn_2$	$C_{30}H_{34}FeN_2O_2Zn$
fw	1293.94	575.81
Crystal size, mm	0.13x0.05x0.04	0.11x0.09x0.08
Crystal system	triclinic	orthorhombic
Space group	P -1	P b c a
a, Å	10.6485(9)	14.1876(4)
b, Å	15.0229(10)	14.5939(3)
c, Å	20.9423(12)	26.9748(5)
α, deg	89.192(5)	90
β, deg	89.647(6)	90
γ, deg	84.943(6)	90
V, $Å^3$	3336.7(4)	5585.2(2)
T, K	173	173
Z	2	8
ρ_{calc}, $g \cdot cm^{-3}$	1.288	1.370
μ, mm^{-1} (Mo1Kα)	4.593	5.438
F (000)	1368	2400
Absoption correction	multi-scan 0.76983- 1.00000	multi-scan 0.94440-1.00000
θ range, deg	3.607-73.847	4.523-73.663
No. of rflns measd	12730	10742
R_{int}	0.0544	0.0658
No. of rlfns unique	12730	5419
No. of params/restraints	765/0	333/0
R1 (I > 2σ(I))	0.0542	0.0410
R1 (all data)	0.0927	0.0600
wR2 (I > 2σ(I))	0.1197	0.0905
wR2 (all data)	0.1438	0.1040
Diff. Fourier peaks min/max, $eÅ^{-3}$	-0.590/1.433	-0.365/0.468
CCDC number	2297408	2297407

Table S1. Crystal data, data collection and refinement parameters for the structures **2a + 3a** and **2b**. Data were collected using a Xcalibur PX Ultra A diffractometer, and the structures were solved and refined using the OLEX2 and SHELX-2019 program systems. 98% Completeness to 0.84 Å resolution.

- X-ray structure of complex **2b:** the crystal structure of **2b** has one independent molecule in the asymmetric unit, crystallizing in the orthorhombic space group Pbca. All non-hydrogen atoms were refined anisotropically. Hydrogen atoms were included in the model at geometrically calculated positions and refined using a riding model.

- X-ray structure of complexes **2a** and **3a**: two independent molecules were found in this crystal structure. One molecule of compound **2a** and **3a** crystallize together in the triclinic space group P-1. All non-hydrogen atoms were refined anisotropically. Hydrogen atoms were included in the model at geometrically calculated positions and refined using a riding model, except the two bridging hydrides of compound **3a** which have been determined from the Fourier map and refined freely. Carbon atoms of one of the Cp rings (C3, C4, C5, C7, C8) were found to be slightly disordered and were modelled by using simu and delu restraints.

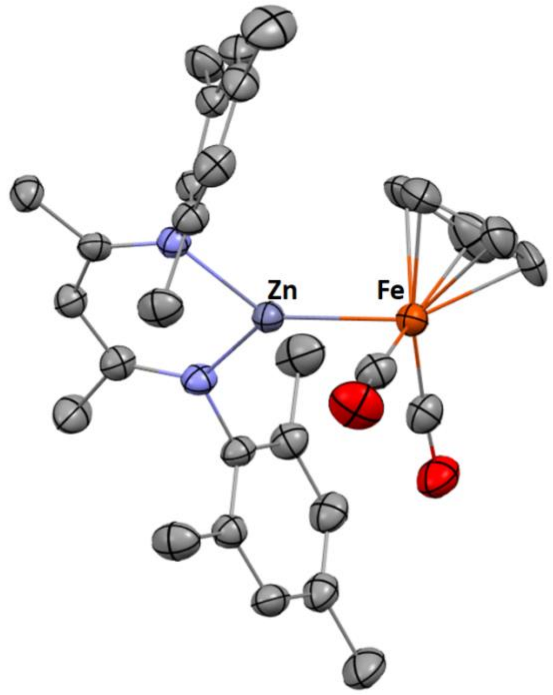

Figure S19. Crystal structure of **2b**. Thermal ellipsoids are shown at a 50 % probability level. Hydrogens atoms have been hidden for clarity.

6. DFT calculations

Density Functional Theory (DFT) calculations were run with Gaussian 09 (Revision D.01)[10] using the BP86[11] functional and an ultrafine integrations grid (keyword int=ultrafine). Empirical dispersion was not included in the optimization as it was found to over-estimate binding energies.[12] Metal atoms (Fe, Zn) were described with Stuttgart SDDAll RECPs and associated basis sets, while a hybrid basis set was used for the other atoms: 6-31g**(C, H)/ 6-311+g*(N, O).[13] The basis set for metal hydrides was expanded by adding diffuse and polarisation functions. The graphical user interface used to visualise the various properties was GaussView 6.0.[14] Single point corrections were performed using a higher basis set (def2TZVP) for C, H, N and O atoms and included solvent corrections (benzene, epsilon = 2.2706) which were applied using the polarized continuum model (PCM) to free energies.[15]
Natural Bond Orbital analysis was carried out in NBO 6.0.[16] QTAIM calculations were performed using the AIMAll sotware.[17] ETS-NOCV calculations were performed for complex **3a** using Orca 4.2.1 quantum chemistry software package.[18] Optimised geometries of complex 3a from the Gaussian 09 calculations detailed above were used. Single-point calculations were performed using the wb97x functional on the relevant fragments. The def2-tzvpp basis set was used for all atoms. Graphical surface representations shown below were plotted using Avogadro 1.2.0.TD-DFT calculations[19] were performed on complex **2a** using the same functional and basis set employed for the geometry optimizations to predict UV-VIS spectroscopy data (the two more intense transitions are shown in Table S4).

	**2a**	**2b**	**2c**	**3a**
	NPA charges			
Zn	1.31	1.30	1.31	1.49
Fe	-0.40	-0.39	-0.41	-0.37
H	-	-	-	-0.15/-0.14
	WBI Indeces			
Zn—Fe	0.31	0.32	0.31	0.12
Zn–H	-	-	-	0.15/0.14
Fe–H	-	-	-	0.53/0.54

Table S2. NBO data for complexes **2a**, **2b, 2c** and **3a**.

[10] M. J. Frisch, G. W. Trucks, H. B. Schlegel, G. E. Scuseria, M. A. Robb, J. R. Cheeseman, G. Scalmani, V. Barone, B. Mennucci, G. A. Peterson, Gaussian 09; Revision D.01, Gaussian Inc. **2009**.
[11] D. Becke, Phys. Rev. A, **1988**, *38*, 3098–3100; J. P. Perdew, Phys. Rev. B, **1986**, *33*, 8822–8824.
[12] T. Husch, L. Freitag, M. Reiher, *J. Chem. Theory Comput.* **2018**, 14, 2456−2468; T. Weymuth, E. P. A. Couzijn, P. Chen, M. Reiher, *J. Chem. Theory Comput.* **2014**, *10*, 3092−3103.
[13] W. J. Hehre, R. Ditchfield, J. A. Pople, *J. Chem. Phys*. **1972**, *56*, 2257–2261; P. C. Hariharan, J. A. Pople, *Theor. Chim. Acta*, **1973**, *28*, 213–222; T. Clark, J. Chandrasekar, W. Spitznagel, Gunther, P. von R. Schleyer, *J. Comput. Chem*. **1983**, *4*, 294–301.
[14] R. Dennington, T. Keith, J. Milliam, GaussView 5.0, Semichem Inc., Shawnee Mission, KS., **2009**.
[15] J. Tomasi, B. Mennucci, R. Cammi, *Chem. Rev.* **2005**, *105*, 2999–3093.
[16] E. D. Glendening, J. K. Badenhoop, A. E. Reed, J. E. Carpenter, J. A. Bohmann, C. M. Morales, C. R. Landis, F. Weinhold, NBO 6.0., **2013**; F. Weinhold and C. R. Landis, *Chem. Educ. Res. Pr.* **2001**, *2*, 91–104
[17] T. A. Keith, AIMALL (Version 19.10.12). TK Gristmill Software, Overland PArk KS, USA., **2019**; F. Cortés-Guzmán and R. F. W. Bader, *Coord. Chem. Rev*. **2005**, *249*, 633–662.
[18] F. Neese, F. Wennmohs, U. Becker, C. Riplinger, *J. Chem. Phys*. 2**020**, *152*, 224108; F. Neese, Wiley *Interdiscip. Rev. Comput. Mol. Sci.* **2018**, *8*, DOI 10.1002/wcms.1327.
[19] C. Adamoab, D. Jacquemin, *Chem. Soc. Rev*., **2013**, *42*, 845–856.

	3a	
	X-ray structure	Computed structure
Zn—Fe (Å)	2.4036(9)	2.362
fsr[a]	1.00	0.98
Zn–H (Å)	2.01(8)	1.835
	2.05(8)	1.832
Fe–H (Å)	1.52(8)	1.540
	1.63(9)	1.530
Zn–H–Fe (°)	81(3)	88.5
	85(3)	88.6

Table S3. Structural parameters of **3a** (experimental vs computational data).
[a] *fsr* = d(Zn–Fe)/(r(Zn) + r(Fe)); r = Pauling radii.[20]

TD-DFT calculations on complex 2a: the computed UV-vis spectrum agrees with the experimental UV-vis absorption spectra (maximum absorption at 350 nm). The two more intense transitions (328 and 316 nm) in the computed spectrum correspond to a combination of several transitions (Table S4) between orbitals that are highly delocalized across the structure. Some of them involve transitions from orbitals containing metal-metal bond character (160, 161) to orbitals with metal-metal antibonding character (167) or centred in the Fe fragment (166) or BDI ligand (165). Orbitals 160 to 167 (HOMO-4 to LUMO+2) are represented in Figure S20.

**Excited state 13**		**Excited State 18**	
3.7730 eV; 328.61 nm; f=0.0578		3.9196 eV; 316.32 nm; f=0.0582	
transitions	coefficients	transitions	coefficients
159 ->165	-0.19632	158 ->165	0.28448
160 ->165	-0.26761	160 ->165	-0.11288
160 ->167	-0.13833	160 ->166	-0.22510
161 ->165	-0.10860	161 ->166	-0.11522
161 ->166	0.18911	162 ->167	-0.30253
161 ->167	0.11284	162 ->169	0.10616
162 ->167	0.15125	163 ->167	-0.12274
163 ->165	0.10933	164 ->165	-0.15350
164 ->165	-0.23549	164 ->170	0.32298
164 ->169	0.39982	164 ->171	0.11375
		164 ->172	0.14907

Table S4. TD-DFT calculations. Excited states 13 and 18 are the two more intense transitions in the calculated UV-vis spectrum.

[20] R. J. Eisenhart, L. J. Clouston, C. C. Lu, *Acc. Chem. Res.* **2015**, *48*, 2885–2894.

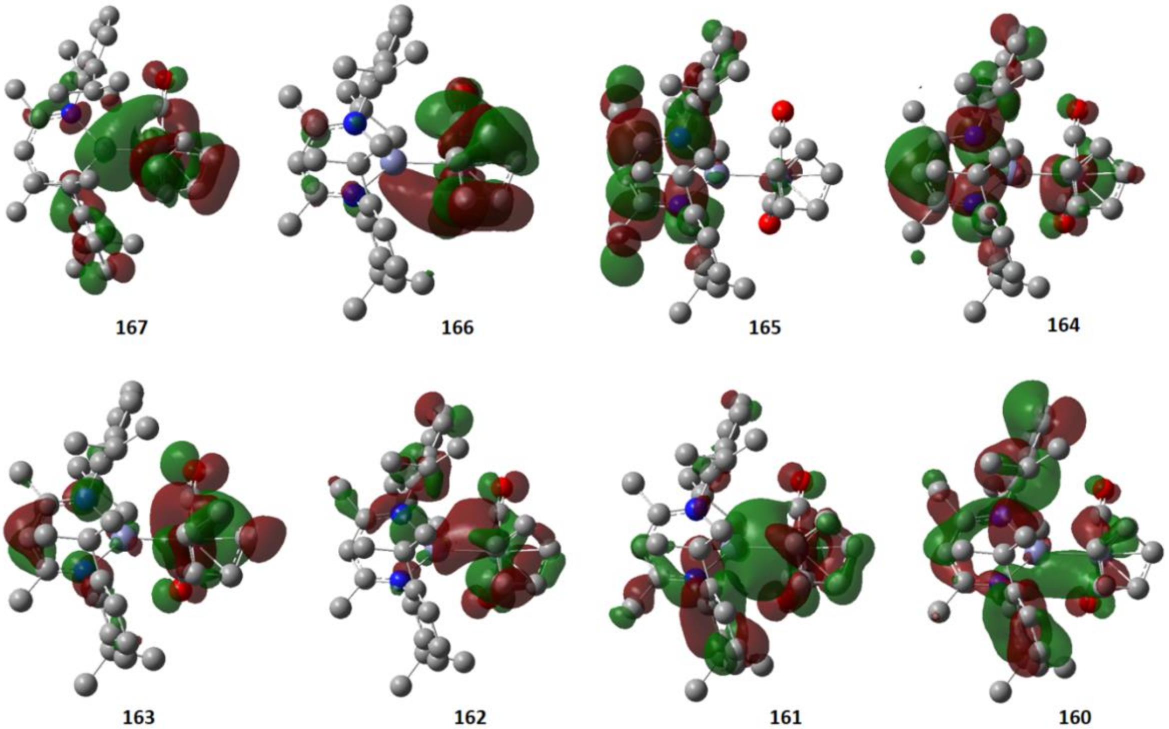

Figure S20. Representation of orbitals 160 to 167 (HOMO-4 to LUMO+2) from complex **2a**.

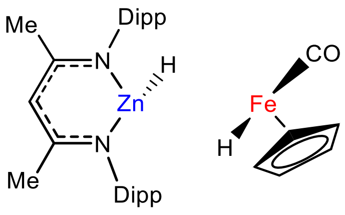	**ΔE_{orb}**	-80.4	
	$\Delta\rho_1$	-41.1	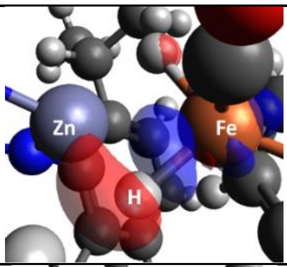
	$\Delta\rho_2$	-25.8	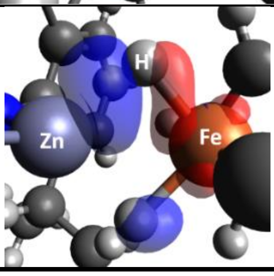
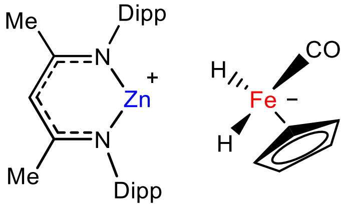	**ΔE_{orb}**	-93.5	
	$\Delta\rho_1$	-43.6	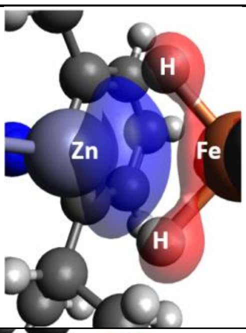
	$\Delta\rho_2$	-15.2	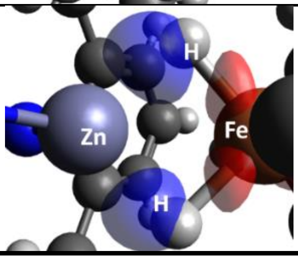
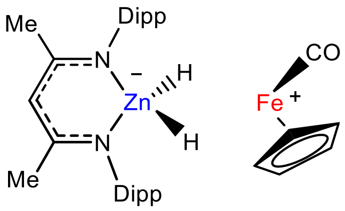	**ΔE_{orb}**	-146.9	
	$\Delta\rho_1$	-75.1	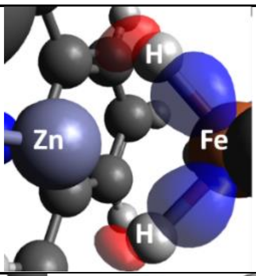
	$\Delta\rho_2$	-35.0	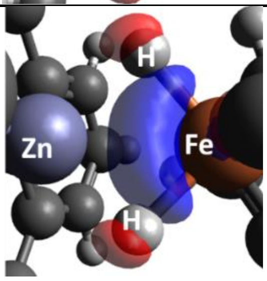

Table S5. ETS-NOCV data (in kcal/mol) computed for complex **3a** fragmentation. Selected deformation density plots. Charge flow from red to blue.

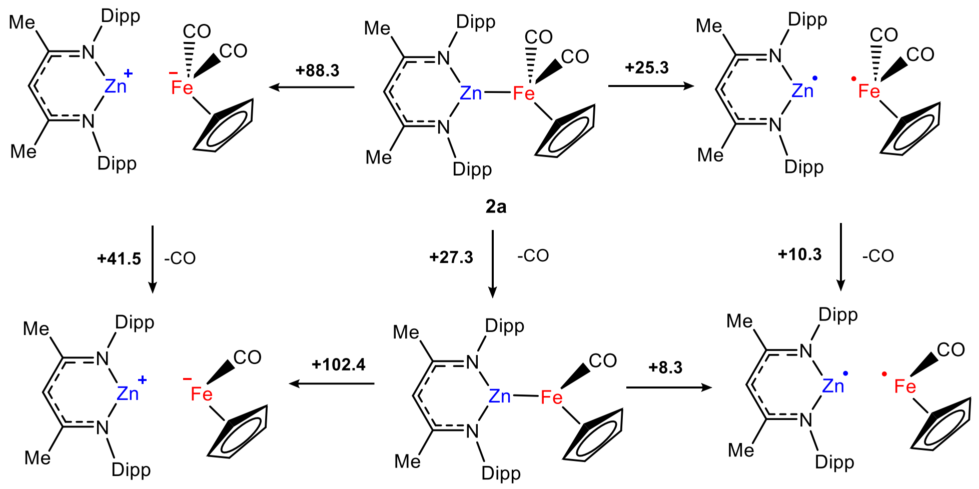

Figure S21. CO and metal-metal bond dissociation Gibbs Free Energies at 298K in kcal mol-1. G09: M062X / def2TZVP (C,H,N,O) / SDDAll (Fe,Zn) // M062X / 6-31g**(C, H) / 6-311+g*(N, O) / SDDAll (Fe,Zn). Solvent corrections (benzene, epsilon = 2.2706) which were modelled using the polarized continuum model (PCM).

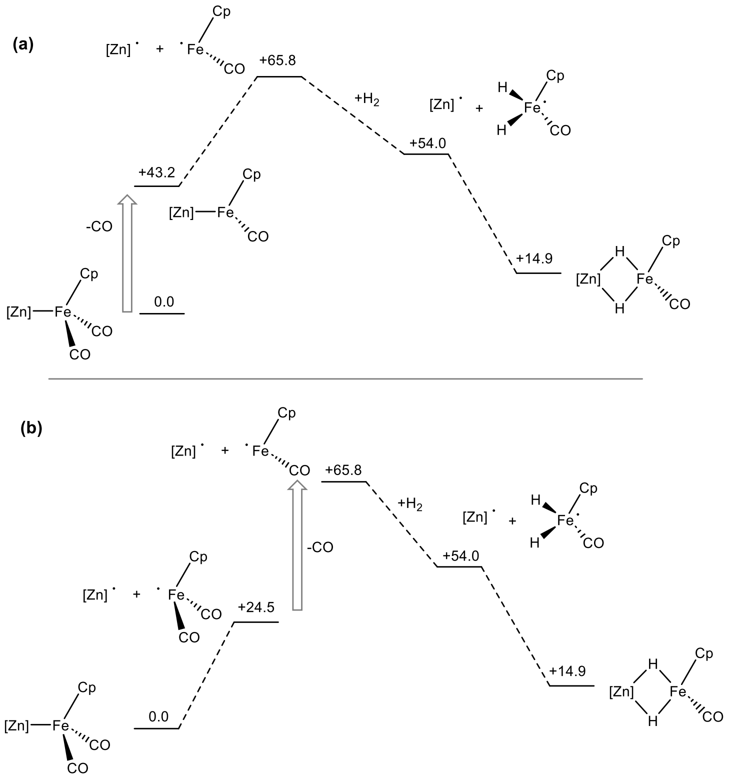

Figure S22. Thermodynamic profile for homolytic dissociation of the metal-metal bond and oxidative addition in the Fe radical species. **(a)** CO dissociation prior metal-metal homolysis and **(b)** CO dissociation after metal-metal bond cleavage. G09: BP86 / def2TZVP (C,H,N,O) / SDDAll (Fe,Zn) // BP86/ 6-31g**(C, H) / 6-311+g*(N, O) / SDDAll (Fe,Zn). Solvent corrections (benzene, epsilon = 2.2706) which were modelled using the polarized continuum model (PCM).

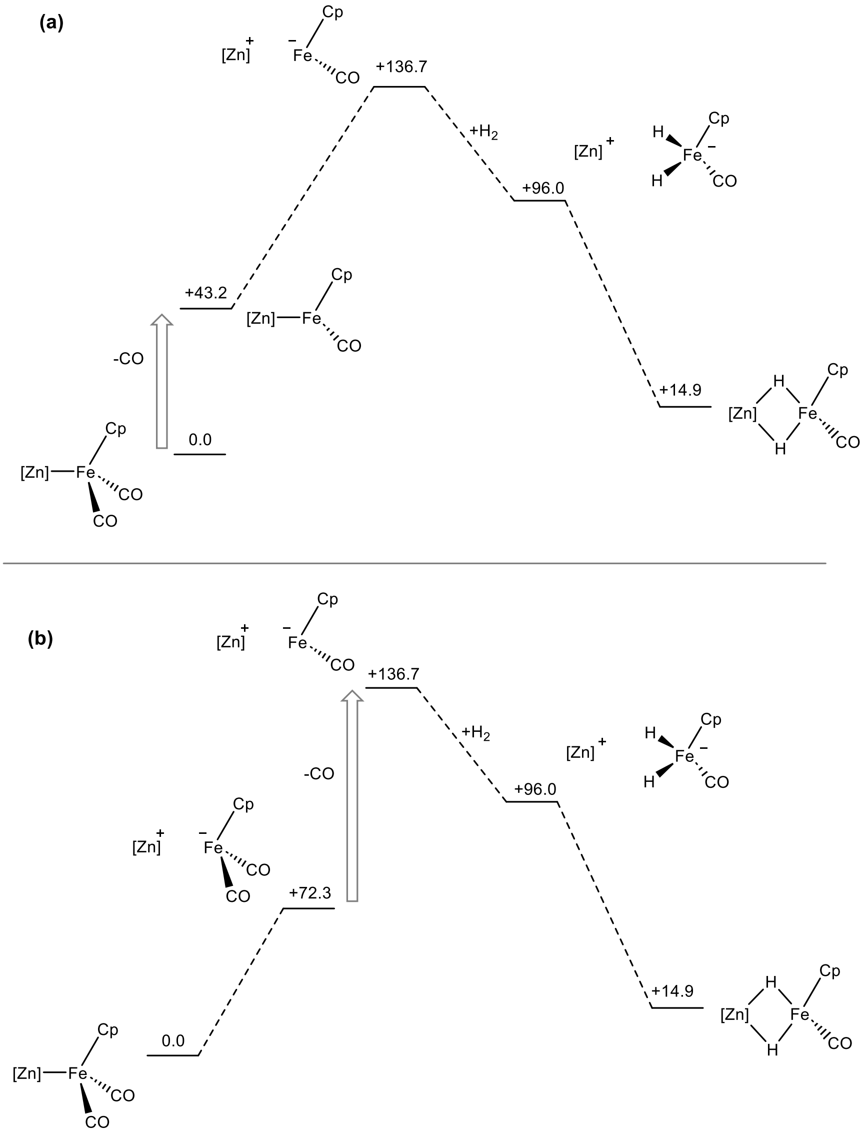

Figure S23. Thermodynamic profile for heterolytic dissociation of the metal-metal bond and oxidative addition in the Fe anion species. **(a)** CO dissociation prior metal-metal heterolysis and **(b)** CO dissociation after metal-metal bond cleavage. G09: BP86 / def2TZVP (C,H,N,O) / SDDAll (Fe,Zn) // BP86/ 6-31g**(C, H) / 6-311+g*(N, O) / SDDAll (Fe,Zn). Solvent corrections (benzene, epsilon = 2.2706) which were modelled using the polarized continuum model (PCM).

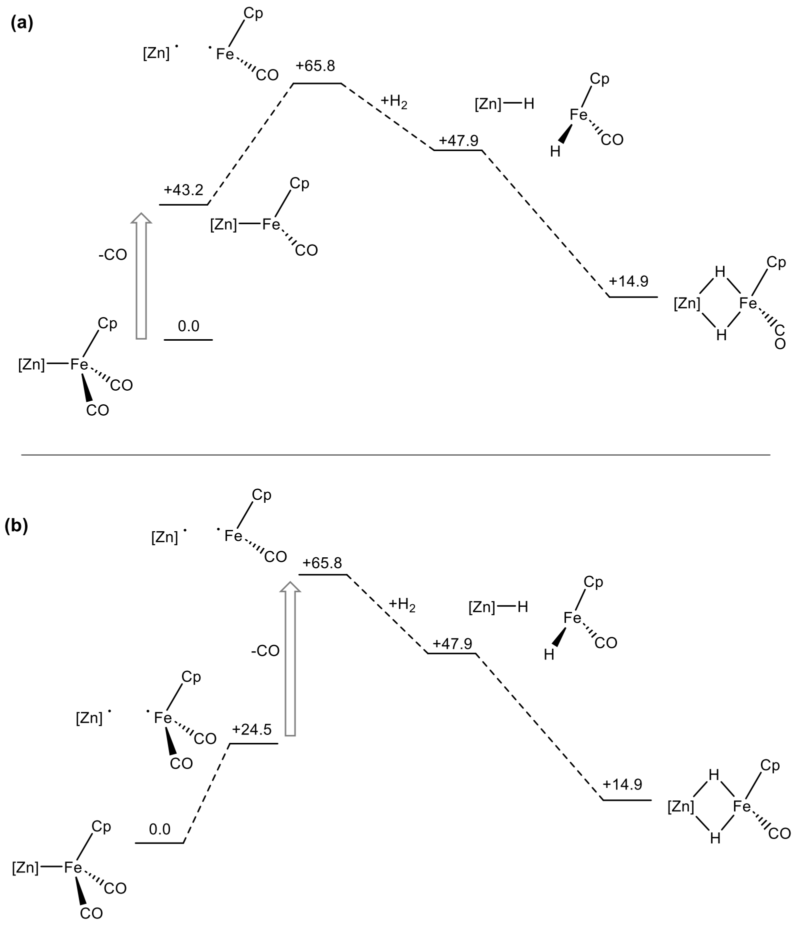

Figure S24. Thermodynamic profile for homolytic dissociation of the metal-metal bond and H_2 activation forming **1a** and 'CpFe(H)(CO)' intermediates. **(a)** CO dissociation prior metal-metal homolysis and **(b)** CO dissociation after metal-metal bond cleavage. G09: BP86 / def2TZVP (C,H,N,O) / SDDAll (Fe,Zn) // BP86/ 6-31g**(C, H) / 6-311+g*(N, O) / SDDAll (Fe,Zn). Solvent corrections (benzene, epsilon = 2.2706) which were modelled using the polarized continuum model (PCM).

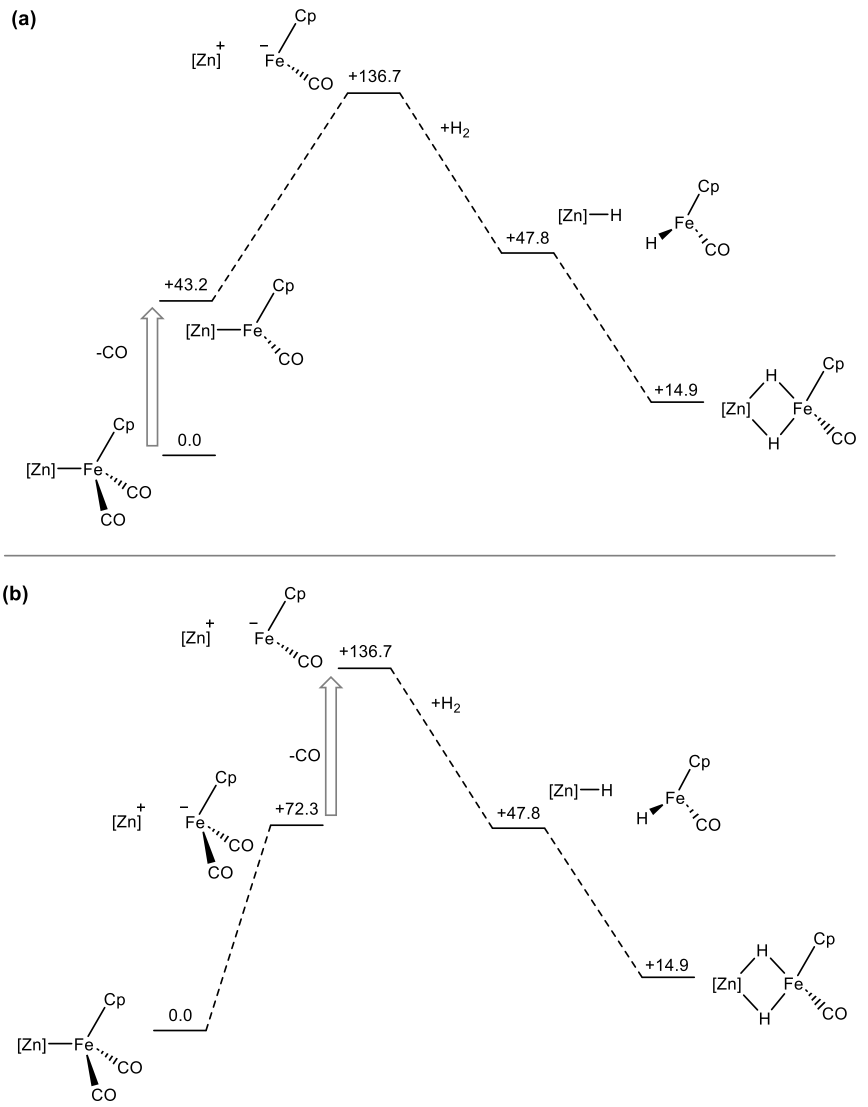

Figure S25. Thermodynamic profile for heterolytic dissociation of the metal-metal bond and H_2 activation forming **1a** and 'CpFe(H)(CO)' intermediates. **(a)** CO dissociation prior metal-metal heterolysis and **(b)** CO dissociation after metal-metal bond cleavage. G09: BP86 / def2TZVP (C,H,N,O) / SDDAll (Fe,Zn) // BP86/ 6-31g**(C, H) / 6-311+g*(N, O) / SDDAll (Fe,Zn). Solvent corrections (benzene, epsilon = 2.2706) which were modelled using the polarized continuum model (PCM).

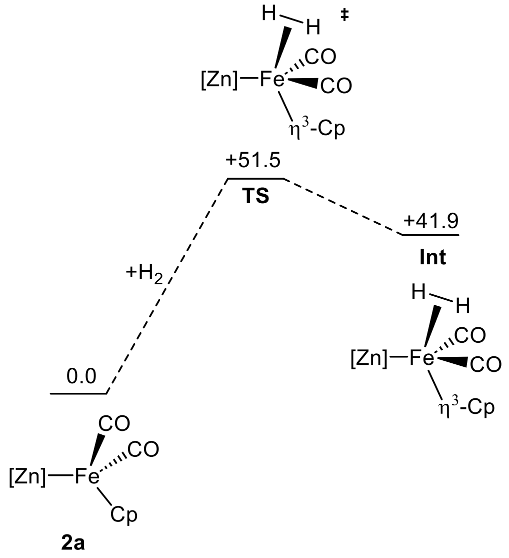

Figure S26. Thermodynamic profile for an associative mechanism where the H_2 coordinates to the Fe centre prior dissociation of the CO. G09: BP86 / def2TZVP (C,H,N,O) / SDDAll (Fe,Zn) // BP86/ 6-31g**(C, H) / 6-311+g*(N, O) / SDDAll (Fe,Zn). Solvent corrections (benzene, epsilon = 2.2706) which were modelled using the polarized continuum model (PCM).

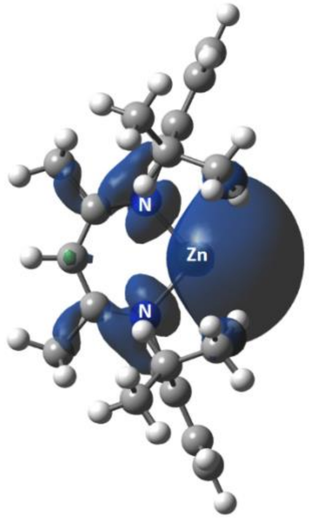

Figure S27. Natural Spin Density plot of [Zn] radical species formed after metal-metal bond homolytic cleavage. NPA (Zn) = 0.86; NSP (Zn) = 0.77.

7. XYZ coordinates

2a-CO.log

Zn	2.057527	9.867672	5.130853
Fe	3.348732	11.708799	5.942897
N	1.378104	8.786063	3.566687
N	1.550439	8.429420	6.561076
C	0.334681	6.836046	2.451798
H	-0.303101	7.513289	1.860886
H	-0.250708	5.948872	2.735243
H	1.150121	6.509862	1.784294
C	1.481889	9.394982	2.265636
C	1.024906	6.119201	7.291041
H	1.929311	6.091300	7.922235
H	0.874023	5.126169	6.842203
H	0.177113	6.324405	7.966347
C	1.156628	7.190760	6.217032
C	3.403059	13.059312	7.587553
H	4.130219	13.029468	8.400927
C	0.880102	7.541352	3.685696
O	4.910712	11.814727	3.497448
C	0.699947	11.037089	0.643847
H	-0.024454	11.801384	0.340179
C	3.604738	7.985573	1.760739
H	3.267318	7.478300	2.681090
C	0.539635	10.394298	1.886613
C	1.814561	9.715056	10.612189
H	1.883407	10.071820	11.645170
C	-1.986137	10.281180	2.150503
H	-2.166666	10.768573	1.176013
H	-2.836084	10.524745	2.812341
H	-1.987794	9.190830	1.983351
C	2.681345	9.733609	0.174775
H	3.515129	9.488061	-0.492287
C	1.635832	8.793184	7.943924
C	2.563755	9.050991	1.400648
C	0.445990	9.104087	8.671366
C	1.763519	10.718882	-0.207982
H	1.876690	11.235256	-1.167040
C	0.565605	9.565100	9.997359
H	-0.342299	9.814002	10.558327
C	-0.653361	10.760586	2.772152
H	-0.526672	10.229810	3.733010
C	0.837784	6.804603	4.894050
H	0.479978	5.777273	4.789626
C	2.912445	8.938931	8.565532
C	2.975230	9.399300	9.894520
H	3.951140	9.509766	10.378770
C	3.555854	13.768371	6.363917
H	4.408894	14.380528	6.071189
C	2.137787	12.360925	7.564546

H	1.742083	11.725392	8.356721
C	1.505025	12.640792	6.308929
H	0.508461	12.327252	6.000895
C	2.389245	13.491786	5.551871
H	2.207360	13.873765	4.547232
C	4.202586	8.655574	7.795624
H	3.952403	7.953786	6.978678
C	-0.949280	8.997208	8.047203
H	-0.849447	8.490219	7.072753
C	4.982207	8.618615	2.063152
H	5.382439	9.137322	1.174321
H	5.709244	7.839788	2.355407
H	4.918903	9.361719	2.872205
C	-1.924277	8.166902	8.912790
H	-1.522264	7.165596	9.143359
H	-2.885396	8.034356	8.386363
H	-2.142404	8.664628	9.873975
C	3.749353	6.912541	0.656128
H	2.782160	6.452915	0.389225
H	4.428705	6.108830	0.989783
H	4.175907	7.339944	-0.268229
C	4.272924	11.691326	4.481841
C	4.730240	9.961149	7.138421
H	3.907189	10.154627	6.344548
H	5.664649	9.799355	6.578525
H	4.875285	10.757368	7.879576
C	-1.541817	10.399062	7.773918
H	-1.662764	10.969745	8.711667
H	-2.534013	10.318450	7.295927
H	-0.887684	10.985303	7.108054
C	5.314237	8.005924	8.645512
H	5.706315	8.692565	9.415816
H	6.164341	7.718726	8.003541
H	4.947698	7.099091	9.155283
C	-0.709803	12.274396	3.072687
H	0.235079	12.625956	3.519101
H	-1.533147	12.501118	3.773087
H	-0.885745	12.865123	2.156580

2a.log

Zn	2.151052	9.904902	5.124976
Fe	3.253709	12.021242	5.299899
N	1.520883	8.786512	3.557860
N	1.645471	8.557442	6.577415
C	0.506675	6.778084	2.511689
H	-0.510314	7.140971	2.277037
H	0.439621	5.700262	2.722592
H	1.112756	6.934221	1.606752
C	1.475178	9.381576	2.245622
C	1.080538	6.266952	7.366142
H	2.060556	5.830903	7.628807

H	0.429867	5.445424	7.030605
H	0.664168	6.702053	8.286778
C	1.258185	7.304211	6.263929
C	4.089656	13.140852	6.895352
H	5.149913	13.135779	7.149831
C	1.063266	7.531557	3.712246
O	2.789362	12.617272	2.494433
C	0.264833	10.603843	0.525116
H	-0.642716	11.109747	0.177692
C	3.939871	8.693018	1.881706
H	3.899492	8.632917	2.984416
C	0.283981	10.029381	1.810017
C	1.831133	9.826902	10.639713
H	1.875629	10.165552	11.680187
O	5.616090	10.390636	4.827482
C	-2.259888	9.767614	2.000175
H	-2.509934	10.477049	1.192024
H	-3.101446	9.777059	2.714682
H	-2.203395	8.760686	1.552355
C	2.552254	9.917729	0.133543
H	3.429434	9.887301	-0.521022
C	1.714209	8.950101	7.959504
C	2.633169	9.342222	1.416532
C	0.587950	9.616389	8.538509
C	1.381926	10.540081	-0.315939
H	1.344731	10.985667	-1.315637
C	0.673994	10.039575	9.878636
H	-0.179417	10.549349	10.336253
C	-0.945322	10.150287	2.714310
H	-0.810690	9.451987	3.558880
C	1.011932	6.847397	4.950262
H	0.677790	5.809307	4.884000
C	2.897382	8.730637	8.723886
C	2.927813	9.180481	10.060242
H	3.832405	9.014902	10.656343
C	3.456578	14.022485	5.967737
H	3.937599	14.814630	5.394631
C	3.096984	12.232415	7.413101
H	3.262869	11.441415	8.143609
C	1.839696	12.555793	6.800368
H	0.884592	12.080074	7.009214
C	2.061639	13.654465	5.904828
H	1.308752	14.124954	5.271908
C	4.134305	8.031299	8.153697
H	3.904871	7.739254	7.113958
C	-0.698343	9.853805	7.737072
H	-0.397612	9.999157	6.680927
C	5.183574	9.526363	1.503102
H	5.369061	9.513902	0.414301
H	6.079891	9.110745	1.993340
H	5.080280	10.577333	1.816604

C	-1.640623	8.625018	7.770923
H	-1.179754	7.736414	7.314549
H	-2.571279	8.841116	7.217161
H	-1.914565	8.376440	8.811679
C	4.092360	7.248643	1.347026
H	3.280363	6.589734	1.694969
H	5.048127	6.811469	1.685889
H	4.087796	7.238293	0.242279
C	2.954633	12.300840	3.609964
C	5.355654	8.978719	8.117616
H	5.144505	9.888122	7.534512
H	6.222268	8.476633	7.654311
H	5.650412	9.285156	9.137258
C	-1.487606	11.102386	8.187295
H	-1.972211	10.946726	9.167455
H	-2.291290	11.319342	7.463361
H	-0.852357	12.000406	8.271761
C	4.493408	6.752633	8.947461
H	4.806771	6.997763	9.977718
H	5.332436	6.223226	8.463561
H	3.643587	6.053892	9.020698
C	-1.043077	11.575874	3.308050
H	-0.130695	11.832796	3.873670
H	-1.910637	11.660871	3.986970
H	-1.161186	12.327322	2.507549
C	4.634870	11.010544	5.015885

3a.log

Zn	6.195753	2.463255	5.299008
N	5.613496	1.135414	3.911918
Fe	6.670620	4.818083	5.185458
C	6.642827	1.695936	8.134444
N	6.256669	1.186222	6.841683
C	4.962827	-1.102865	3.092408
H	4.012316	-0.766589	2.644286
H	4.830216	-2.127625	3.468474
H	5.704117	-1.120718	2.275399
C	8.018305	1.704424	8.504656
C	9.115806	1.159114	7.587011
H	8.624406	0.726117	6.698166
C	5.952242	-0.115859	6.709632
C	10.050891	2.290338	7.098486
H	9.484953	3.074722	6.569522
H	10.817142	1.889757	6.410834
H	10.575112	2.766635	7.945749
C	7.876888	1.016091	2.023403
H	7.755815	0.489811	2.986046
C	5.422705	1.618095	2.571405
C	6.025585	-1.023088	7.927732
H	7.049114	-1.046378	8.338334
H	5.723552	-2.050168	7.676605

H	5.376509	-0.649392	8.737060
C	3.643208	3.709490	8.492003
H	3.717919	4.252655	9.450589
H	2.581367	3.711490	8.187184
H	4.221724	4.271365	7.740377
C	9.936379	0.036234	8.262415
H	10.489766	0.413035	9.140461
H	10.677403	-0.377720	7.556254
H	9.295210	-0.793528	8.606271
C	2.929335	2.053775	3.101429
H	3.211359	1.442511	3.976095
C	6.527340	1.647694	1.671180
C	4.164532	2.259897	8.628732
H	4.059131	1.773104	7.643313
C	4.154664	2.144112	2.187792
C	4.036763	2.753507	0.923040
H	3.070518	3.172488	0.619984
C	6.058393	2.763283	10.240685
H	5.304267	3.181988	10.916654
C	8.978286	2.082451	2.219328
H	9.126671	2.675371	1.298959
H	9.941719	1.604717	2.470419
H	8.715942	2.773711	3.037074
C	5.401884	-0.162411	4.203314
C	8.370870	2.247420	9.754927
H	9.425934	2.263625	10.050662
C	5.649168	2.233906	9.001810
C	8.314452	-0.026915	0.969357
H	7.543248	-0.800510	0.812950
H	9.242689	-0.530650	1.290645
H	8.515221	0.443353	-0.009302
C	5.560787	-0.723076	5.490211
H	5.348772	-1.792456	5.555988
C	1.741854	1.353297	2.401840
H	1.364891	1.945405	1.549457
H	0.904216	1.221143	3.108583
H	2.022218	0.358419	2.015525
C	6.354679	2.269184	0.418712
H	7.199304	2.309801	-0.278433
C	3.299298	1.469009	9.637101
H	3.336026	1.924098	10.642529
H	3.633569	0.421916	9.734753
H	2.243131	1.458950	9.315119
C	5.126780	2.831712	0.046207
H	5.015720	3.316243	-0.929550
C	2.503501	3.441811	3.631119
H	3.315938	3.906783	4.213402
H	1.619298	3.353312	4.286733
H	2.240117	4.121463	2.800879
C	7.405008	2.771416	10.621768
H	7.701840	3.191690	11.588375

C	6.264346	5.194334	3.136877
H	5.931238	4.463867	2.400585
C	6.252883	6.841158	4.784190
H	5.912152	7.556624	5.532751
C	7.112222	5.067078	6.842448
O	7.428459	5.343174	7.937089
C	7.629710	6.543921	4.456095
H	8.509901	6.995891	4.913573
C	7.628906	5.531239	3.447233
H	8.514136	5.079925	3.000363
C	5.421164	6.007835	3.973553
H	4.332154	5.983476	3.997187
H	7.624939	3.604101	5.093249
H	5.381518	4.081877	5.618964

Fe-anion-CO.log

Fe	-0.226160	0.405595	0.277451
C	1.283931	0.110906	-0.485688
O	2.340116	-0.099459	-1.031729
C	-1.574560	0.690763	-1.189763
H	-1.323840	0.999813	-2.206580
C	-1.599487	-0.689768	-0.705697
H	-1.370280	-1.573846	-1.304162
C	-2.192175	0.705314	1.031203
H	-2.409007	1.064259	2.043723
C	-2.012374	-0.682553	0.675680
H	-2.215175	-1.546373	1.310161
C	-1.971966	1.560813	-0.110958
H	-2.139763	2.637664	-0.156978

Fe-anion.log

Fe	3.566970	12.068126	5.199510
C	3.846681	13.159399	7.008862
H	4.800695	13.227694	7.533965
O	3.233659	11.709714	2.328757
O	5.730897	10.116411	5.242829
C	3.386639	14.043610	5.982195
H	3.896728	14.934082	5.614386
C	2.849845	12.130499	7.210774
H	2.929684	11.299651	7.913356
C	1.767177	12.369229	6.309002
H	0.852800	11.781937	6.221863
C	2.106797	13.544001	5.539870
H	1.500968	13.973464	4.740034
C	3.378299	11.848999	3.506546
C	4.842754	10.914823	5.216111

Fe-radical-CO.log

Fe	3.391483	11.913283	5.164408
C	3.892980	13.155381	6.857317
H	4.898986	13.265581	7.264108

O	3.764987	12.324028	2.291845
C	3.318304	13.941255	5.813393
H	3.797821	14.766723	5.287644
C	2.925078	12.163128	7.247094
H	3.064144	11.402797	8.017822
C	1.731009	12.365339	6.468149
H	0.816804	11.773558	6.529159
C	1.971483	13.449284	5.571146
H	1.273255	13.844445	4.833548
C	3.635138	12.106730	3.439057

Fe-radical.log

Fe	3.389290	11.891084	5.176782
C	3.894434	13.159941	6.841967
H	4.900337	13.265697	7.247451
O	3.797284	12.331560	2.316154
O	5.544076	9.933704	5.440753
C	3.314234	13.947094	5.811673
H	3.792206	14.769233	5.279593
C	2.918572	12.162730	7.244425
H	3.061282	11.407374	8.017191
C	1.727881	12.373917	6.481983
H	0.819977	11.771493	6.533066
C	1.973817	13.444566	5.567241
H	1.269098	13.839467	4.835463
C	3.621965	12.128432	3.453360
C	4.666139	10.695348	5.320960

Zn-cation.log

Zn	1.607167	9.473697	5.226669
N	1.524291	8.669659	3.518280
N	1.151659	8.311903	6.640064
C	0.526616	6.739254	2.317391
H	0.334365	7.461017	1.509739
H	-0.385220	6.151908	2.504059
H	1.302826	6.040113	1.961706
C	1.801494	9.396182	2.315144
C	0.698024	5.946405	7.192171
H	1.456002	6.019703	7.989050
H	0.780202	4.963950	6.706565
H	-0.288659	6.005931	7.682252
C	0.878914	7.060635	6.181767
C	0.961034	7.435565	3.592723
C	1.445789	11.416409	1.001124
H	0.965955	12.387195	0.853362
C	3.652935	7.708515	1.624510
H	3.250514	7.151859	2.487789
C	1.173450	10.672938	2.162991
C	1.590732	9.456565	10.698523
H	1.714616	9.749292	11.745631
C	-1.139668	10.545228	3.255003

H	-1.661890	10.764604	2.308291
H	-1.762110	10.925811	4.082286
H	-1.061784	9.450921	3.347710
C	2.989530	9.717471	0.227376
H	3.714711	9.372808	-0.516585
C	1.271356	8.666164	8.029643
C	2.770128	8.935238	1.379305
C	0.123192	8.756372	8.862545
C	2.323825	10.929948	0.023421
H	2.509112	11.513310	-0.883525
C	0.320163	9.145625	10.203457
H	-0.546254	9.217354	10.868547
C	0.241965	11.234493	3.237219
H	0.730728	11.017684	4.253452
C	0.741987	6.726150	4.807628
H	0.393168	5.702937	4.646007
C	2.568192	9.027075	8.502937
C	2.704545	9.410597	9.847829
H	3.691315	9.671482	10.240640
C	3.760783	9.050673	7.542308
H	3.630695	8.220146	6.822904
C	-1.299739	8.553768	8.336616
H	-1.238427	8.089593	7.336820
C	5.082795	8.161952	2.011390
H	5.557919	8.721493	1.187638
H	5.716639	7.287441	2.236752
H	5.074736	8.818108	2.898768
C	-2.151397	7.629118	9.234256
H	-1.663189	6.655397	9.409438
H	-3.131644	7.441012	8.765344
H	-2.344813	8.083150	10.220876
C	3.699239	6.745024	0.417656
H	2.691722	6.433208	0.095665
H	4.273680	5.839501	0.675189
H	4.196067	7.206932	-0.452313
C	3.785067	10.379464	6.744715
H	2.785429	10.661649	6.286034
H	4.535705	10.379124	5.937077
H	3.973919	11.242537	7.406862
C	-1.994044	9.926348	8.156106
H	-2.093102	10.449375	9.122640
H	-3.005753	9.796798	7.735320
H	-1.421812	10.584974	7.479512
C	5.133734	8.849815	8.215445
H	5.400179	9.691986	8.876146
H	5.925890	8.768752	7.452846
H	5.144197	7.926738	8.816784
C	0.094510	12.766366	3.220885
H	1.070125	13.277838	3.198565
H	-0.458736	13.104310	4.112183
H	-0.485480	13.092300	2.340619

Zn-radical.log

Zn	1.750483	9.839654	5.207037
N	1.288290	8.723725	3.584099
N	1.414576	8.422568	6.611129
C	0.565285	6.655584	2.448304
H	-0.250693	7.142711	1.887496
H	0.262309	5.626147	2.689043
H	1.432542	6.619018	1.767013
C	1.388855	9.342559	2.290708
C	0.770879	6.165668	7.373992
H	1.689264	6.007399	7.964811
H	0.446095	5.199489	6.961061
H	0.003457	6.523954	8.081379
C	1.007150	7.186603	6.271007
C	0.899919	7.441761	3.707144
C	0.424284	10.723424	0.534615
H	-0.425414	11.277330	0.120069
C	3.861040	8.611807	2.171793
H	3.523069	7.994246	3.022228
C	0.273211	10.054293	1.764409
C	2.083186	9.634284	10.630822
H	2.261254	9.966960	11.658960
C	-2.269304	9.780507	1.642139
H	-2.413043	10.503432	0.820047
H	-3.193473	9.783082	2.245970
H	-2.160274	8.779757	1.190127
C	2.728877	10.004239	0.370349
H	3.680525	9.996198	-0.172692
C	1.624947	8.777391	7.987972
C	2.631999	9.317870	1.596019
C	0.560296	9.360371	8.733052
C	1.637730	10.700603	-0.163281
H	1.734331	11.228225	-1.118129
C	0.816191	9.778978	10.052952
H	0.006609	10.232066	10.635983
C	-1.053201	10.145257	2.521237
H	-1.013290	9.419542	3.352233
C	0.776684	6.755721	4.940905
H	0.448276	5.717773	4.851342
C	2.921267	8.632215	8.559776
C	3.122768	9.067574	9.883672
H	4.116168	8.964619	10.334502
C	4.095168	8.064743	7.759484
H	3.686529	7.632260	6.829563
C	-0.823772	9.582317	8.120200
H	-0.857113	9.031967	7.163734
C	4.868728	9.646793	2.725216
H	5.233726	10.311037	1.921781
H	5.742660	9.143305	3.175220
H	4.401582	10.282992	3.498044

C	-1.964778	9.041196	9.009033
H	-1.823532	7.973332	9.248669
H	-2.935693	9.149851	8.495014
H	-2.036634	9.590176	9.964338
C	4.542402	7.670550	1.154872
H	3.834345	6.928193	0.748375
H	5.371890	7.123201	1.635527
H	4.968427	8.226780	0.301509
C	5.070100	9.194972	7.353498
H	4.553532	9.973842	6.764821
H	5.901491	8.797411	6.744832
H	5.502885	9.682340	8.245166
C	-1.039237	11.079909	7.798512
H	-0.999896	11.690898	8.717782
H	-2.022592	11.242438	7.322750
H	-0.258446	11.456040	7.113723
C	4.840352	6.938753	8.508825
H	5.333564	7.311088	9.423841
H	5.626460	6.505396	7.866204
H	4.156787	6.125296	8.806675
C	-1.225438	11.550771	3.143779
H	-0.386271	11.791672	3.820388
H	-2.163888	11.612133	3.722822
H	-1.256651	12.328607	2.360250

8. References

1. M. Garçon, N. W. Mun, A. J. P. White, M. R. Crimmin, *Angew. Chem. Int. Ed.* **2021**, *60*, 2–11.
2. S. Schulza, T. Eisenmanna, D. Schuchmanna, M. Bolteb, M. Kirchnera, R. Boesea, J. Spielmanna, S. Harder, *Z. Naturforsch.* **2009**, *64b,* 1397–1400.
3. T. J. Mazzacano, N. P. Mankad, *J. Am. Chem. Soc.* **2013**, *135*, 17258–17261.
4. O. V. Dolomanov, L. J. Bourhis, R. J. Gildea, J. A. K. Howard, H. Puschmann, *J. Appl. Crystallogr*. **2009**, 42, 339–341; SHELX-2013, G.M. Sheldrick, *Acta Cryst*. **2015**, C71, 3–8.
5. NaFp was prepared by reduction of Fp_2 following the literature procedures: M. E. Giuseppetti, A. R. Cutler, *Organometallics* **1987**, *6*, 970−973.
6. ^{Mes}(BDI)Zn–I was prepared according to the literature procedures: S. Schulz, T. Eisenmann, U. Westphal, S. Schmidt, U. Flörke, *Z. Anorg. Allg. Chem*. **2009**, *635*, 216−220.
7. NaFp' was prepared by reduction Fp'_2 following the same method as for NaFp.
8. ^{Dep}(BDI)Zn–Cl was prepared according to the literature procedures: M. Cheng, D. R. Moore, J. J. Reczek, B. M. Chamberlain, E. B. Lobkovsky, G. W. Coates, *J. Am. Chem. Soc.* **2001**, *123*, 8738−8749.
9. Higher pressure of H_2 were also employed (4 bar) and although lower yields for the by-products Fp_2 and **1a** were obtained (<10 %), lower conversions were observed and the yield for **3a** could not be improved.
10. M. J. Frisch, G. W. Trucks, H. B. Schlegel, G. E. Scuseria, M. A. Robb, J. R. Cheeseman, G. Scalmani, V. Barone, B. Mennucci, G. A. Peterson, Gaussian 09; Revision D.01, Gaussian Inc. **2009**.
11. D. Becke, Phys. Rev. A, **1988**, *38*, 3098–3100; J. P. Perdew, Phys. Rev. B, **1986**, *33*, 8822–8824.
12. T. Husch, L. Freitag, M. Reiher, *J. Chem. Theory Comput.* **2018**, *14*, 2456−2468; T. Weymuth, E. P. A. Couzijn, P. Chen, M. Reiher, *J. Chem. Theory Comput*. **2014**, *10*, 3092−3103.
13. W. J. Hehre, R. Ditchfield, J. A. Pople, *J. Chem. Phys*. **1972**, *56*, 2257–2261; P. C. Hariharan, J. A. Pople, *Theor. Chim. Acta*, **1973**, *28*, 213–222; T. Clark, J. Chandrasekar, W. Spitznagel, Gunther, P. von R. Schleyer, *J. Comput. Chem*. **1983**, *4*, 294–301.
14. R. Dennington, T. Keith, J. Milliam, GaussView 5.0, Semichem Inc., Shawnee Mission, KS., **2009**.
15. J. Tomasi, B. Mennucci, R. Cammi, *Chem. Rev.* **2005**, *105*, 2999–3093.
16. E. D. Glendening, J. K. Badenhoop, A. E. Reed, J. E. Carpenter, J. A. Bohmann, C. M. Morales, C. R. Landis, F. Weinhold, NBO 6.0., **2013**; F. Weinhold and C. R. Landis, *Chem. Educ. Res. Pr.* **2001**, *2*, 91–104.
17. T. A. Keith, AIMALL (Version 19.10.12). TK Gristmill Software, Overland PArk KS, USA., **2019**; F. Cortés-Guzmán and R. F. W. Bader, *Coord. Chem. Rev*. **2005**, 249, 633–662.
18. F. Neese, F. Wennmohs, U. Becker, C. Riplinger, *J. Chem. Phys*. **2020**, *152*, 224108; F. Neese, Wiley *Interdiscip. Rev. Comput. Mol. Sci.* **2018**, *8*, DOI 10.1002/wcms.1327.
19. C. Adamoab, D. Jacquemin, *Chem. Soc. Rev*., **2013**, *42*, 845–856.
20. R. J. Eisenhart, L. J. Clouston, C. C. Lu, *Acc. Chem. Res.* **2015**, *48*, 2885–2894.
